# Proton Tautomerism for Anhydrous Superprotonic Conduction in 1,2,3‐Triazolium Dihydrogen Phosphate Crystal

**DOI:** 10.1002/anie.202520785

**Published:** 2026-02-01

**Authors:** Kaito Nishioka, Shun Dekura, Tomoko Fujino, Motohiro Mizuno, Reiji Kumai, Bo Thomsen, Motoyuki Shiga, Yuta Hori, Yasuteru Shigeta, Hatsumi Mori

**Affiliations:** ^1^ The Institute for Solid State Physics The University of Tokyo Kashiwa Chiba Japan; ^2^ Institute of Multidisciplinary Research for Advanced Materials Tohoku University Sendai Japan; ^3^ Department of Chemistry and Life Science Yokohama National University Yokohama Kanagawa Japan; ^4^ Nanomaterials Research Institute Kanazawa University Kanazawa Ishikawa Japan; ^5^ Department of Chemistry Graduate School of Natural Science & Technology Kanazawa University Kanazawa Ishikawa Japan; ^6^ Institute for Frontier Science Initiative Kanazawa University Kanazawa Ishikawa Japan; ^7^ Graduate University for Advanced Studies SOKENDAI Tsukuba Japan; ^8^ Photon Factory Institute of Materials Structure Science High Energy Accelerator Research Organization (IMSS, KEK) Tsukuba Ibaraki Japan; ^9^ Center For Computational Science & e‐Systems Japan Atomic Energy Agency Chiba Japan; ^10^ Center for Computational Sciences University of Tsukuba Tsukuba Ibaraki Japan; ^11^ Institute of Philosophy in Interdisciplinary Sciences Kanazawa University Kanazawa Ishikawa Japan

**Keywords:** acid–base single crystal, anhydrous proton conduction, hydrogen‐bond, isotropic superprotonic conduction, proton tautomerism

## Abstract

Proton dynamics within molecular organic solids are crucial for energy‐related technologies. Proton conductors for use as solid electrolytes in hydrogen fuel cells have been developed, elucidating the higher proton transport mechanism and establishing design guidelines for higher conduction. Many anhydrous proton conductors for proton transport utilizing molecular motion in solids have been studied; however, low‐barrier conduction is challenging. In this study, we addressed proton tautomerism as a new guideline for proton conduction, rather than molecular motion. The key to facilitating low‐barrier conduction is proton transport without molecular motion via dynamic interconversion between multiple tautomers. We demonstrated the effectiveness of proton‐tautomerism strategy in 1,2,3‐triazole dihydrogen phosphate crystal, which exhibited low‐barrier, isotropic superprotonic conductivity exceeding 10^−3^ S cm^−1^. Both theoretical and experimental results confirmed that superprotonic conduction originates from proton tautomerism, demonstrating for the first time that proton tautomerism can serve as a design guide for highly efficient anhydrous proton conductors.

## Graphical abstract

This study investigates the contribution of proton tautomerism to solid‐state proton conduction and achieves isotropic superprotonic conduction in acid–base single crystals. Specifically, low‐barrier isotropic superprotonic conductivity exceeding 10^−3^ S cm^−1^ is achieved in a cocrystal of 1,2,3‐triazole and phosphoric acid. We demonstrate proton tautomerism as a new guideline for anhydrous proton conduction, as an alternative to molecular motion.

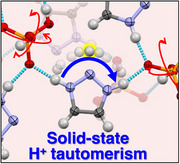

## 1 Introduction

Control of proton dynamics in molecular organic solids is of fundamental scientific interest and essential for anhydrous high‐proton conductors, that is, solid electrolytes for hydrogen fuel cells operable in the medium temperature range above 100°C with no need for humidified environments [1, 2, 3]. However, issues such as dopant leakage, swelling‐induced degradation, and reaction with electrodes remain. To address these problems while maintaining high conductivity and realizing excellent conductors, new solid electrolytes must be developed. A material design guideline based on an understanding of the conduction mechanism is essential. However, given the nonstoichiometric and heterogeneous compositions of doped polymers and porous materials, and their lack of crystallinity, exploration of conduction mechanisms based on structure–property relationships is extremely difficult.

Intrinsic anhydrous proton conductivity has recently been studied using single‐crystal samples to establish design guidelines for achieving high conductivity [3, 4, 5, 6, 7, 8, 9, 10, 11]. Molecular organic single crystals are considered ideal model materials because they have well‐defined structures, and the conduction mechanism is not influenced by interfaces and defects. Previous studies on acid–base‐type imidazolium dicarboxylate single crystals suggested that the anhydrous proton‐conduction mechanism in molecular crystals is based on a Grotthus mechanism [12]; in other words, the conduction mechanism is based on a hydrogen bond network structure involved in a conduction pathway, intermolecular differences in acidity for intermolecular proton transfer, and molecular reorientation preceding intramolecular proton transfer from one side to the other side within one molecule [7, 8]. Therefore, proton conductors for superprotonic conductivity (>10^−4^ S cm^−1^) have been developed based on design strategies of utilizing molecular motion. Although realization of superprotonic conductivity has been reported [3, 6, 11], systematic control of molecular motion in crystals has not been established. Molecular motion‐induced proton conduction has been achieved; nonetheless, in most cases, the high activation energies (*E*_a_ about 2−6 eV) would prevent conductivity enhancement [7, 8, 9, 10]. Therefore, an intramolecular proton transfer mechanism that can be strategically introduced and controllable at the molecular design stage is necessary as an alternative to molecular reorientation.

**FIGURE 1:**
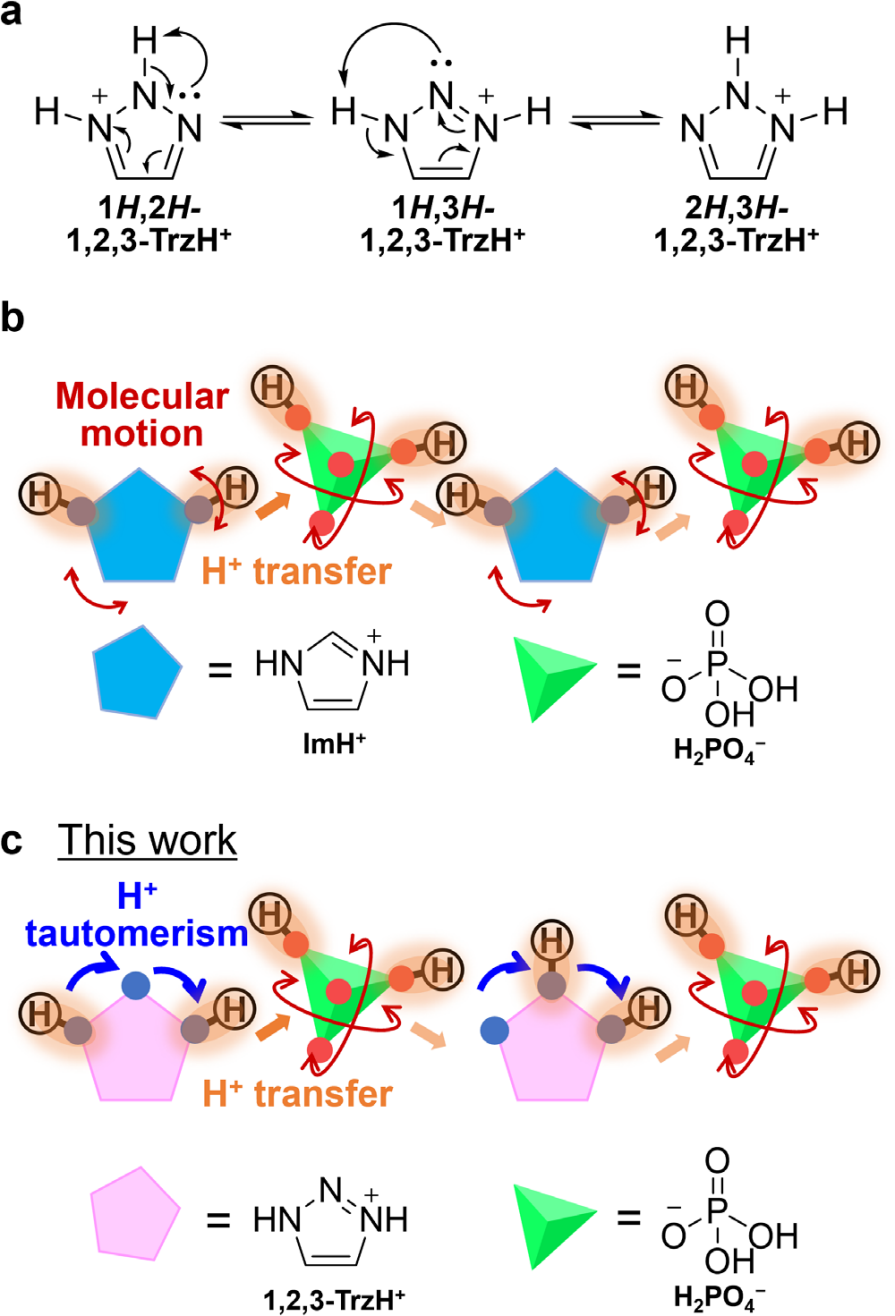
Proton tautomerism and proton conduction based on molecular motion and proton tautomerism. (a) Changes in chemical structures and π‐electronic configuration of **1,2,3‐TrzH^+^**. (b) Rotational motion of **ImH** ^**+**^ and H_2_PO_4_^−^. (c) Proton tautomerism of **1,2,3‐TrzH** ^+^ and rotational motion of dihydrogen H_2_PO_4_^−^.

Proton tautomerism is a phenomenon in which isomers with different intramolecular proton positions undergo rapid interconversion in conjunction with recombination of the π‐electronic structures (Figure 1a). Generally, proton tautomerism is known in organic chemistry as a peculiar molecular property [13, 14, 15]; however, few studies have focused on proton tautomerism in crystals. In conventional conduction mechanisms such as that of imidazolium dihydrogen phosphate (**ImH^+^**)(H_2_PO_4_^−^) [16], when a molecule receives a proton from its left neighbor, molecular rotation (for intramolecular proton transfer) is necessary to pass it to its right neighbor (Figure 1b). However, reorientation‐free intramolecular proton transfer is possible for a molecule exhibiting proton tautomerism, such as 1,2,3‐triazolium (=**1,2,3‐TrzH^+^**; Figure 1a). Unlike molecular motion, proton tautomerism can be incorporated at the molecular design stage, its conduction mechanism can be discussed based on the static average structure, and strategic achievement of low‐barrier long‐distance proton transfer by controlling the molecular arrangement is likely possible. Previous studies reported anhydrous proton conduction in materials containing multiple tautomeric species, particularly **1,2,3‐Trz**, where *E*_a_ was low (∼0.3 eV) [17, 18, 19, 20, 21, 22, 23]. However, in all of the previous studies, the specific role of multiple tautomers in the solid‐state proton conduction remains unexplored experimentally, and the dynamic interconversion of tautomers—which is, in essence, proton tautomerism—has not been demonstrated experimentally to contribute directly to proton conduction. Moreover, the solid‐state proton conductivity of **1,2,3‐Trz** was only ∼3 × 10^−5^ S cm^−1^ because of the poor thermal stability of the neutral crystal. Therefore, proton tautomerism has never been conclusively proven to directly govern proton conduction and has not been considered a practical guideline for realizing high proton conductivity.

This study realized isotropic superprotonic conduction exceeding 10^−3^ S cm^−1^ using proton tautomerism in acid–base single crystals and elucidated the contribution of proton tautomerism to solid‐state proton conduction for the first time. For comparison with a previous study on imidazolium phosphate [16], we consider phosphoric acid and **1,2,3‐Trz** as acid and base molecules, respectively; the former and latter conduct protons through well‐understood isotropic reorientation [24, 25, 26, 27, 28, 29] and unexplored proton tautomerism in a solid, respectively (Figure 1c). Hence, we prepare new single crystals of 1,2,3‐triazolium dihydrogen phosphate (**1,2,3‐TrzH^+^**)(H_2_PO_4_^−^) (=**1**). Despite the anisotropic hydrogen‐bonding network of the crystal structure, an isotropic anhydrous superprotonic conductivity exceeding 10^−3^ S cm^−1^ is achieved at 396 K. This is the first instance of an anhydrous crystal exceeding 10^−3^ S cm^−1^ facilitated by proton tautomerism. Solid‐state ^2^H nuclear magnetic resonance (NMR) spectroscopy using **1,2,3‐TrzH^+^** partially H/D isotopically substituted (**1,2,3‐Trz‐*d*_2_‐H^+^**)(H_2_PO_4_^−^) polycrystals suggests that **1,2,3‐Trz‐*d*_2_‐H^+^** exhibits minor libration motion that does not contribute to conduction. Variable‐temperature single‐crystal x‐ray structural analysis, ab initio nonequilibrium molecular dynamics (NEMD), and density functional theory (DFT) calculations confirm for the first time that the **1,2,3‐TrzH^+^** proton tautomerism can aid the development of superprotonic conductivity in solids.

## 2 Results and Discussion

### 2.1 Thermal Properties

High‐quality single crystals of **1** were newly obtained through dichloromethane vapor diffusion from a mixture of **1,2,3‐Trz** and phosphoric acid in a 1:1 stoichiometric ratio in acetone (see the Supporting Information for details). The thermal stability of these single crystals was evaluated based on melting‐point measurement and thermogravimetry/differential thermal analysis (TG/DTA). Melting occurred at 410 K: an endothermic peak and weight loss were concomitantly observed from the DTA and TG chart, respectively. Thus, sample decomposition, possibly 1,2,3‐Trz evaporation, occurred at approximately the melting point (Figure S7). The phase behavior of single‐crystalline **1** was investigated using differential scanning calorimetry (DSC) below the melting point. From room temperature to 400 K, the DSC chart exhibited no peaks indicative of phase transitions. Overall, **1** was stable in the mid‐temperature range above 100°C (373 K) to ∼400 K with no phase transition. These characteristics support the application of **1** as an anhydrous solid electrolyte for fuel cells.

### 2.2 Single‐Crystal Structure of **1**

**FIGURE 2:**
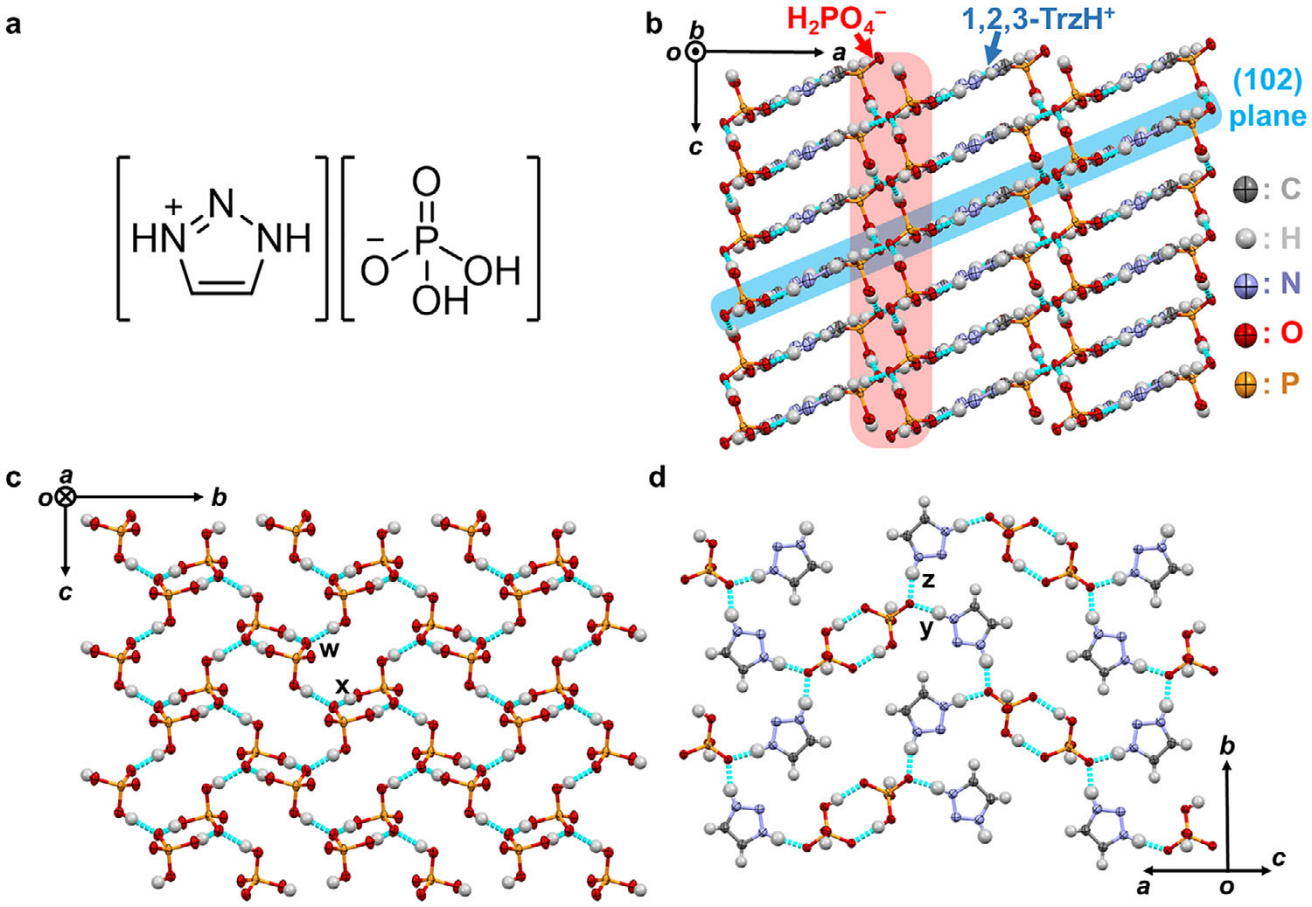
Chemical and crystal structures of 1,2,3‐triazolium dihydrogen phosphate (=**1**). (a) Chemical structure of **1**. (b, c) Novel crystal structure of **1** along *b‐* and *a*‐axes, respectively (*w*, *x*: hydrogen bonds of *d*_O···O_ = 2.573(2), 2.586(2) Å). (d) (102) plane in crystal structure (*y*, *z*: hydrogen bonds of *d*_N···O_ = 2.609(3), 2.661(3) Å). Gray: C, white: H, blue: N, red: O, orange: P. The light‐blue dashed lines correspond to hydrogen bonds.

X‐ray structural analysis at 298 K (Figure 2) revealed the crystal structure of **1** [30]. The space group was monoclinic *P*2_1_/c with crystallographically independent one **1,2,3‐TrzH^+^** and one H_2_PO_4_^−^ molecules in a 1:1 stoichiometric ratio. The unit cell contained four pairs of (**1,2,3‐TrzH^+^**)(H_2_PO_4_^−^). The crystals were anhydrous: free of water and other crystallization solvent molecules (Figure 2a and Table S1). H_2_PO_4_^−^ anions formed a two‐dimensional (2D) hydrogen‐bonding network extending on the *bc* plane with hydrogen‐bond distances *d*_O···O_ = 2.573(2), 2.586(2) Å (Figure 2c). From the hydrogen‐bond distances, the hydrogen‐bond protons between the H_2_PO_4_^−^ anions were in double‐well potentials. No disorder of the hydrogen‐bonded protons was observed. The **1,2,3‐TrzH^+^** cations were in the 2D H_2_PO_4_^−^‐layer stacking direction (//*a*), and the H_2_PO_4_^−^ and **1,2,3‐TrzH^+^** layers alternated in the *a*‐axis direction (Figure 2b). In contrast to the H_2_PO_4_^−^ layer, no hydrogen bonds existed between the **1,2,3‐TrzH^+^** cations in the **1,2,3‐TrzH^+^** layers. Hydrogen bonds were present between the H_2_PO_4_^−^ and **1,2,3‐TrzH^+^** layers, with distances *d*_N···O_ = 2.609(3), 2.661(3) Å, forming a hydrogen‐bond network extending in the (102) plane (Figure 2d). Overall, **1** featured a 3D hydrogen‐bonding network comprising anisotropic molecular arrangements; this is likely an anhydrous proton‐conductive structure in any crystal axial direction.

### 2.3 Single‐Crystal Anhydrous Proton Conductivity of **1**

**FIGURE 3:**
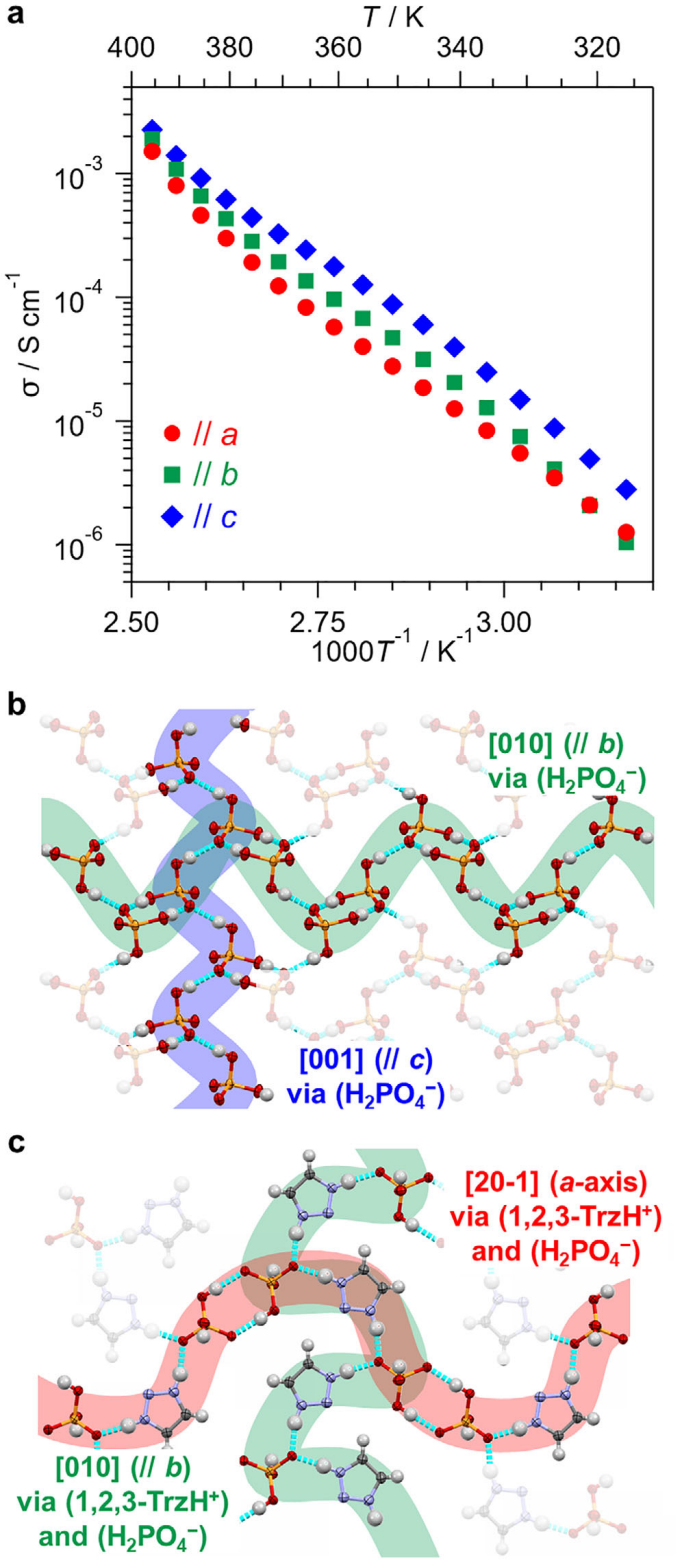
Temperature dependence of anhydrous proton conductivity (*σ*) of **1** and possible conduction pathways. (a) Red circles: along *a*‐axis, green squares: along *b*‐axis, blue diamonds: along *c*‐axis. (b) Pathways along *b*‐ and *c*‐axes in 2D H_2_PO_4_^−^ layer in (100) plane. (c) Pathways along *b*‐axis and [20−1] in (102) plane (gray: C, white: H, blue: N, red: O, orange: P).

To elucidate the intrinsic anhydrous proton conductivity of **1** and its anisotropy, the temperature dependence of anhydrous proton conductivity was measured along each crystallographic axis using alternating‐current (AC) impedance spectroscopy and single‐crystalline samples (see the Supporting Information for details). The crystals were covered with a heat‐resistant epoxy resin to prevent **1,2,3‐Trz** evaporation from the surface during measurement (Figure S9), and the proton conductivity (*σ*) along each axis was evaluated as a function of temperature to ∼400 K (Figure 3a). In all directions, **1** exhibited superprotonic conductivity exceeding 10^−3^ S cm^−1^ at 395.6 K. Protons (H^+^) were identified as the conducting carriers using a fuel‐cell device with single crystals of **1** (Figures S3 and S19). Despite the anisotropic molecular arrangement (Figure 2), the conductivity anisotropy was low. The *E*_a_ estimated from the temperature dependence of *σ* were 0.94(2), 0.98(1), and 0.89(1) eV along the *a*, *b*, and *c* axes, respectively: all <1 eV (Table 1). These *E*_a_ values are considerably lower than those of single‐crystalline salts based on the rotational motions of **ImH^+^** cations (∼2–6 eV)^16^, and comparable to those of other reported phosphate proton conductors [5, 6].

**TABLE 1:** Maximum anhydrous proton conductivity, σ_max_, and activation energy, *E*_a_, of single‐crystal samples of **1** for each crystallographic direction.

	//*a*	//*b*	//*c*
σ_max_/S cm^−1^	1.51 × 10^−3^ [395.6 K]	1.91 × 10^−3^ [395.6 K]	2.26 × 10^−3^ [395.6 K]
*E*_a_/eV	0.94(2) [316.0−395.6 K]	0.97(1) [316.0−395.6 K]	0.89(1) [316.0−395.6 K]

As shown in Figure 2, a 2D hydrogen bonding network of H_2_PO_4_^−^ anions extends in the *bc* plane. In the *b*‐ and *c*‐axis directions, possible proton‐conduction pathways traversed H_2_PO_4_^−^ only (Figure 3b). Because **1,2,3‐TrzH^+^** exists between H_2_PO_4_^−^ in the (102) plane, an alternative proton‐conduction pathway along the *b*‐axis is possible, via both **1,2,3‐TrzH^+^** and H_2_PO_4_^−^ (Figure 3c; green). However, proton conduction along the *a*‐axis is possible only via both **1,2,3‐TrzH^+^** and H_2_PO_4_^−^ (Figure 3c; red). Although the conduction pathways along the respective crystal axes differ (i.e., they are anisotropic), the experimentally observed conductivities and their *E*_a_ values are isotropic (Figure 3a and Table 1). With the isotropic and low *E*_a_ values, these results indicate that proton conduction through **1,2,3‐TrzH^+^** cations is as fast and low‐barrier as that through H_2_PO_4_^−^ anions, possibly due to the proton tautomerism of the **1,2,3‐TrzH^+^** cations. To the best of our knowledge, this is the first example of superprotonic conductivity exceeding 10^−3^ S cm^−1^ in anhydrous crystals exhibiting proton tautomerism.

### 2.4 Molecular Motions of **1,2,3‐TrzH^+^** From Variable‐Temperature Solid‐State ^2^H NMR

**FIGURE 4:**
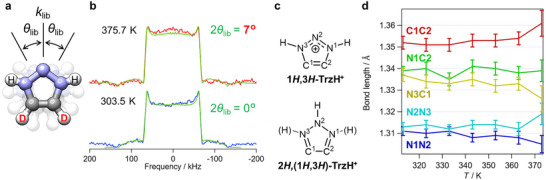
Solid‐state ^2^H NMR of **1‐*d*_2_** and variable‐temperature XRD of **1**. (a) Model of librational motion of **1,2,3‐TrzH^+^** assumed in simulation of solid‐state ^2^H NMR spectra (gray: C, white: H, or D, blue: N). (b) Temperature dependence of ^2^H NMR spectra for **1‐*d*_2_**. The green lines show the simulated powder patterns assuming libration of **1,2,3‐TrzH^+^**. Fixed values of *e* ^2^ *qQ*/*ħ* = 175 kHz and *η* = 0.04 were used for the simulation (see Supporting Information for details). (c) Chemical structures and corresponding atomic labels of **1*H*,3*H*‐TrzH^+^** and **2*H*,(1*H*,3*H*)‐TrzH^+^**. (d) Temperature dependence of bond lengths of **1,2,3‐TrzH^+^** in **1**. The error bars correspond to the estimated standard deviations.

Although the H_2_PO_4_^−^ rotational dynamics contribute to proton conduction, fast and low‐barrier proton conduction via **1,2,3‐TrzH^+^** is possible based on proton tautomerism and molecular rotational motions. To investigate the molecular rotational dynamics of **1,2,3‐TrzH^+^** cations in **1**, we performed variable‐temperature solid‐state ^2^H NMR measurements using a polycrystalline sample of **1**. Solid‐state ^2^H NMR enables investigation of kilohertz‐ to megahertz‐order dynamics, facilitating the evaluation of molecular rotational dynamics in crystals [11, 31, 32]. Here, two C–H protons of **1,2,3‐TrzH^+^** were H/D substituted (=**1,2,3‐Trz‐*d*_2_‐H^+^**; Figure 4a) for selective observation of the cation dynamics only; this did not influence the thermal, structural, and physical properties (Tables S1 and S2, Figures S7 and S15−S18) [30]. Figure 4b shows the solid‐state ^2^H NMR spectra at 303.5 and 375.5 K; with heating, the spectral shape changed only minimally. Spectral simulation assuming librational motion with librational frequency *k*_lib_ and angle *θ*_lib_ reproduced the experimental spectra (Figures 4a andS4). No librational motion was indicated at 303.5 K. Minor librational motion occurred at 375.7 K with *k*_lib_ of 10^8^ Hz and 2*θ*_lib_ of 7°. Although the anhydrous proton conductivity increased by approximately three orders of magnitude, from below 10^−6^ to ∼10^−3^ S cm^−1^, 2*θ*_lib_ was only 7° at 375.5 K, suggesting little molecular motion. In our previous work, **Im‐*d*_3_‐H^+^** cations in (**Im‐*d*_3_‐H^+^**)(H_2_PO_4_^−^) salt exhibited librational motion with 2*θ*_lib_ = 46° at a maximum temperature of 357 K; the conductivity reached ∼10^−5^ S cm^−1^. [16] In contrast, the **1,2,3‐Trz‐*d*_2_‐H^+^** cations in **1‐*d*_2_** had considerably lower kinetics with conductivity more than two orders higher. As evident from the NMR results, the rotational (librational) dynamics of **1,2,3‐TrzH^+^** contribute minimally to the high conductivity in **1**, with the fast proton conduction through **1,2,3‐TrzH^+^** being achieved via proton tautomerism.

### 2.5 Temperature Dependence of the Bond Lengths in **1,2,3‐TrzH^+^**

To obtain direct experimental evidence of the proton tautomerism of **1,2,3‐TrzH^+^** cations in **1**, we performed variable‐temperature single‐crystal X‐ray structural analyses at 313–373 K. [30] The crystal system and space groups were temperature‐independent, consistent with the peak‐free DSC result; the crystal axis length and unit cell volume increased monotonically (Table S3). The room‐temperature structural analysis suggested that **1*H*,3*H*‐TrzH^+^** was the most stable tautomer in **1** (Figure 2); thus, thermally activated protons will appear at the 2*H* site of the **1,2,3‐TrzH^+^** cations at high temperature under proton tautomerism (Figure 4c). However, no 2*H* protons were observed in the high‐temperature structures, possibly due to the low sensitivity of x‐ray to hydrogen and the low ratio of the 2*H*‐tautomer. The tautomer ratio was alternatively evaluated from the bond lengths of the **1,2,3‐TrzH^+^** five‐membered ring, because the proton tautomerism involves π‐electron reconstruction (Figure 4c).

The theoretical bond‐length changes for the **1,2,3‐TrzH^+^** proton tautomerism were estimated using DFT calculations. The N^1^–N^2^, N^2^–N^3^, and C^1^–C^2^ bonds elongate, and the N^1^–C^2^ and N^3^–C^1^ bonds contract when the **1*H*,3*H*‐TrzH^+^** tautomer converts to the **2*H*,(1*H*,3*H*)‐TrzH^+^** tautomer via proton tautomerism (Figure S20; see also the Experimental section in the Supporting Information). Hence, we experimentally investigated the temperature dependence of the bond lengths in the **1,2,3‐TrzH^+^** five‐membered ring. Evident temperature dependencies of the bond‐length changes were noted for N^2^–N^3^, N^3^–C^1^, and C^1^–C^2^, with N^2^–N^3^ and C^1^–C^2^ extending and N^3^–C^1^ detracting with increasing temperature (Figure 4d). However, N^1^−N^2^ exhibited counterintuitive behavior. These changes correspond to the theoretically predicted increase in the **2*H*,(1*H*,3*H*)‐TrzH^+^** proportion due to proton tautomerism. Therefore, an increased fraction of **2*H*,(1*H*,3*H*)‐TrzH^+^** tautomer in the single crystal of **1** with temperature was observed experimentally, suggesting that proton tautomerism contributes to the proton‐conduction mechanism in **1**.

### 2.6 Ab Initio Calculations for Proton Conduction in **1**

To investigate the responsibility of **1,2,3‐TrzH^+^** proton tautomerism for proton conduction, we performed ab initio NEMD simulations (Figures S22–S24). No molecular rotational motion or tautomerism of **1,2,3‐TrzH^+^** was observed for simulations of a perfect crystal; however, proton transfer with H_2_PO_4_^−^ rotation was observed for models with excess protons or proton defects. No large‐angle rotational motion of **1,2,3‐TrzH^+^** was observed, even for those doped models. The calculated perfect‐crystal proton conductivity was ∼10^−5^ S cm^−1^ in all three axial directions (see the Experimental section in the Supporting Information for details); the conductivity of the crystal with excess protons or proton defects was ∼10^−3^ S cm^−1^ in all three axial directions, agreeing with experimental AC impedance measurements. Thus, proton conduction likely occurs near proton excess or defect sites rather than in perfect crystals.

To elucidate the proton‐conduction pathway involving proton tautomerism in **1,2,3‐TrzH^+^** and its *E*_a_, nudged elastic band (NEB) method using DFT calculations was performed. The proton conduction was in the −*b*‐axis direction, along which proton conduction through **1,2,3‐TrzH^+^** proton tautomerism and intermolecular proton transfer without H_2_PO_4_^−^ rotational motion is possible. In the NEB calculations, we constructed three structural models: a‐ perfect crystal and crystals with one proton excess and one defect site introduced in the unit cell, respectively. Two proton‐conduction pathways were found in perfect crystals (Figures S25 and S26), with *E*_a_ ≈ 2 eV, significantly exceeding the experimentally estimated *E*_a_ (∼1 eV). This suggests that the presence of excess or deficient protons is necessary, consistent with the NEMD simulations.

**FIGURE 5:**
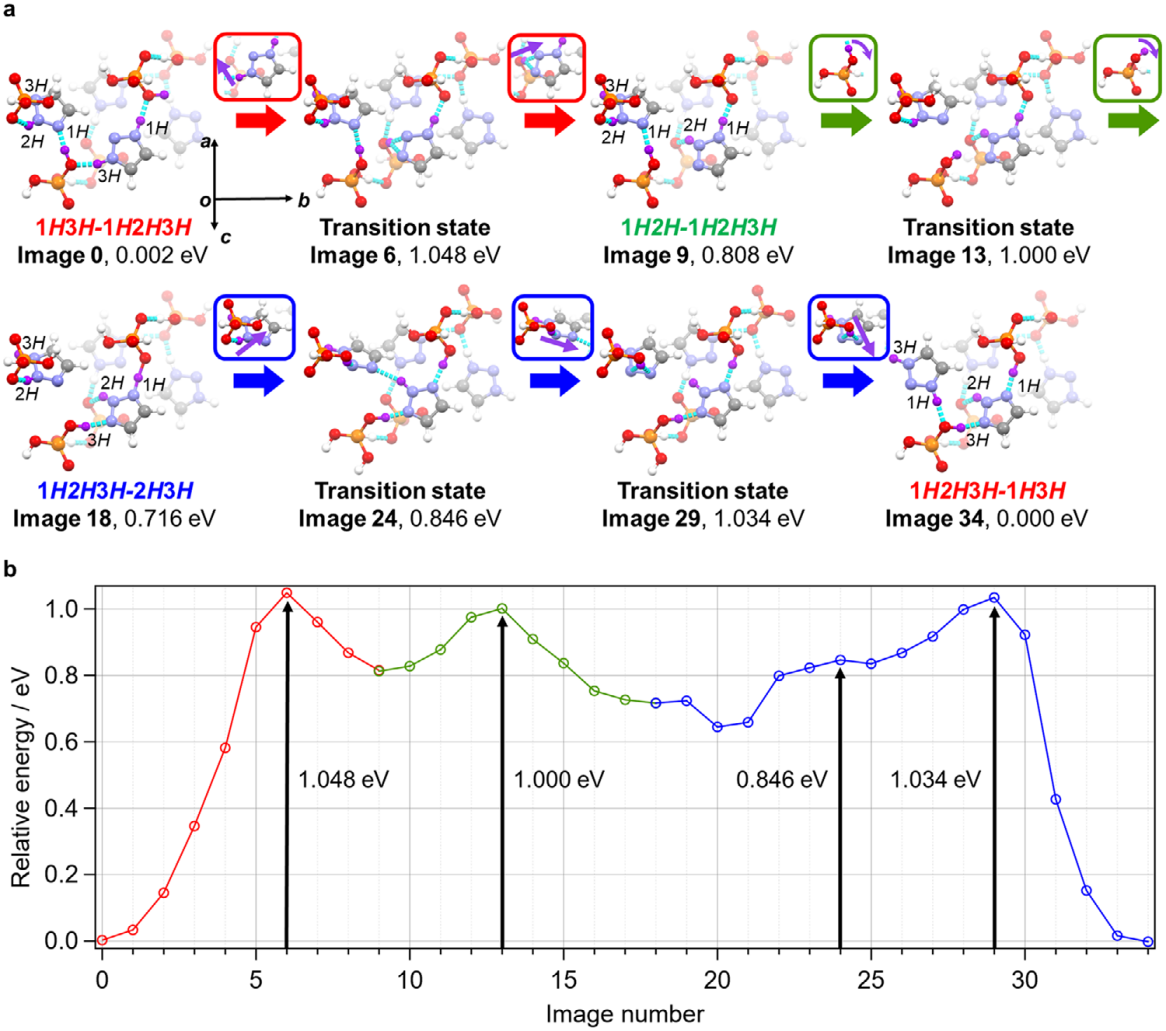
Ab initio NEB calculation of proton tautomerism of **1**. (a) Structures of local minimum states and transition states (gray: C, white: H, blue: N, red: O, orange: P; the conducting protons are colored purple). NEB calculations were performed for each of the three proton transfer divisions. Images 0, 9, 18, and 34 were optimized individually and correspond to the initial or final states of the respective proton transfer divisions. Images 6, 13, 24, and 29 are the transition states. The corresponding image numbers and relative potential energies (the lowest value for Image 34 was set to 0) are shown beneath the structures. (b) Relative energy curve obtained from NEB calculation for tautomerism process of **1** (red, green, and blue: the first, second, and third divisions, respectively).

The relative energy diagram obtained from NEB calculations for the models with proton excess or deficient sites reproduced the experimental *E*_a_ values, unlike the perfect‐crystal case. Figure 5a shows the suggested conduction pathway along the −*b* direction based on the obtained minimum‐energy paths for the proton excess‐site case. We first assumed the conduction pathway along the −*b* direction through four types of metastable structures and optimized these structures independently under the periodic boundary condition of the single unit cell. The structure containing **2*H*‐1,2,3‐Trz** with a proton on the 2*H* position (**1*H*3*H*–1*H*2*H*3*H***; the label does not indicate which proton is attached between the acid or base molecules) was the initial structure, followed by **1*H*2*H*–1*H*2*H*3*H*** and **1*H*2*H*3*H*–2*H*3*H***, to the final state of **1*H*2*H*3*H*–1*H*3*H***. The proton was transferred to the crystallographically equivalent **1,2,3‐TrzH^+^**; this can be repeated to produce a long‐range conduction pathway. The optimized metastable structures were used as the initial or final states. The intermediate states and their relative energies were determined by three NEB calculation processes as the minimum energy paths. The intermediate structures obtained from the NEB calculations (Images 0–34, Figure 5a) visualized the tautomerization process from the initial to final state. Interestingly, the **1,2,3‐TrzH^+^** tautomerization was achieved via transient proton transfer from **1,2,3‐TrzH^+^** to H_2_PO_4_^−^ in the adjacent layer (Images 6, 24, and 29, Figure 5a). The protons then returned from H_3_PO_4_ to **1,2,3‐Trz**, and proton transfer occurred from **1*H*,2*H*‐TrzH^+^** to H_2_PO_4_^−^, forming **2*H*‐1,2,3‐Trz** (Images 9 and 34, Figure 5a). The estimated *E*_a_ values of the proton conduction along the −*b* direction involving the **1,2,3‐TrzH^+^** proton tautomerism were estimated to be 1.048 eV (Figure 5b), consistent with experimental results (∼1 eV; Table 1). Similarly, for the structural model with proton defects, the *E*_a_ of the **1,2,3‐TrzH^+^** proton tautomerism was consistent with the experimental value (Figure S27). The **1,2,3‐TrzH^+^** proton tautomerism requires H_2_PO_4_^−^ assistance because of the high barrier for a single molecule in vacuum (∼2.5 eV; Figure S21). Thus, the super‐protonic conduction in **1** realized in this study is specific to a condensed matter system; the crystal environment (e.g., the molecular arrangements and hydrogen‐bonding network structures) is important. These calculations strongly indicate that the fast proton conduction in **1** is realized through **1,2,3‐TrzH^+^** proton tautomerism.

## 3 Conclusion

In **1**, two possible proton‐conduction pathways exist: one through H_2_PO_4_^−^ alone and the other through both **1,2,3‐TrzH^+^** and H_2_PO_4_^−^. The proton conduction through H_2_PO_4_^−^ is well understood, occurring via H_2_PO_4_^−^‐anion reorientational motion [25, 26, 27]. The latter alternative can occur via the pathways proposed by our theoretical calculations (Figures 5 and S27). Such proton conduction comprises the following steps: 1) hydrogen‐bond reconstruction with H_2_PO_4_^−^ rotation; 2) intermolecular proton transfer between H_2_PO_4_^−^ and **1,2,3‐TrzH^+^**; and 3) **1,2,3‐TrzH^+^** proton tautomerism assisted by H_2_PO_4_^−^ in the adjacent (102) layer (Figure 5a). Proton conduction via proton tautomerism is supported by the following findings: The overall **1,2,3‐TrzH^+^** molecular motion revealed by solid‐state ^2^H NMR was almost zero, the temperature dependence of the bond lengths revealed changes in proportion of the tautomers with temperature, and the NEB‐calculated *E*_a_ assuming proton tautomerism of **1,2,3‐TrzH^+^** was up to 1.048 eV. These findings distinguish this work from previous reports [17, 18, 19, 20, 21, 22, 23], in which the dynamic interconversion of tautomers had not been experimentally verified.

It is worth nothing that, in this study, the proton conductivity is analyzed assuming Arrhenius behavior. Some crystalline materials involving molecular motions are known to exhibit non‐Arrhenius behavior [8, 11, 16]. Nevertheless, in **1**, the temperature dependence of the proton conductivity looks follow Arrhenius behavior, and the *E*_a_ determined from the Arrhenius fit is consistent with the *E*_a_ estimated by first‐principles calculations. Thus, an Arrhenius‐type analysis is justified in this study. Still, we cannot conclude that such a new conduction mechanism in this system is physically interpreted with an Arrhenius equation, which remains an issue for future investigation.

The combined experimental and theoretical results indicate that the isotropic anhydrous superprotonic conduction in **1** primarily stems from proton tautomerism, in addition to the reorientational motion of phosphate anions. Because proton conduction can occur without involving the entire molecular motion, low‐barrier conduction becomes possible. Moreover, changes in the electronic structure associated with proton tautomerism are observable as bond‐length variations. Thus, proton tautomerism can facilitate a new development strategy for efficient anhydrous superprotonic conductors.

For the first time, we experimentally confirmed that the microscopic proton tautomerism in a single molecule can induce a macroscopic crystalline property, namely, superprotonic conduction. Combining the conventional design guideline of increasing molecular motion with thermal‐stability improvement is challenging; however, proton tautomerism can be exploited with effective material design for thermal‐stability improvement; for example, by increasing molecular weights. Therefore, proton tautomerism is advantageous for the realization of highly proton‐conductive materials operable at high temperatures. Use of proton tautomerism in not only molecular crystals but also metal complex crystals and crystalline polymers is expected to enable the production of outstanding energy materials.

## Author Contributions

K.N., S.D., and H.M. conceived the idea and designed the study. K.N. performed the experiments and the NEB calculations and analyzed the results. T.F. helped the synthesis of **1,2,3‐Trz‐*d*_2_**. M.M. developed the simulation software for the solid‐state ^2^H NMR spectra. R.K. contributed to the XRD experiments by advising on their design and interpretation. B.T. and M.S. performed the NEMD calculations and analyzed the results. Y.H. and Y.S. contributed to the NEB calculations by advising on their design and interpretation. K.N., S.D., and H.M. wrote and refined the paper. H.M. supervised the project. All authors discussed the results.

## Conflicts of Interest

The authors declare no conflict of interest.

## Supporting information




**Supporting File 1**: The authors have cited additional references within the Supporting Information [1–27].

**Supporting File 2**: anie71356‐sup‐0002‐Data.zip.

## Data Availability

The data that support the findings of this study are available from the corresponding author upon reasonable request.
