# Supplementary material for: Proton Tautomerism for Anhydrous Superprotonic Conduction in 1,2,3‐Triazolium Dihydrogen Phosphate Crystal
Source: Angew Chem Int Ed Engl. 2026 Feb 1;65(11):e20785. doi: 10.1002/anie.202520785 (PMC12970496; doi:10.1002/anie.202520785)
Supplement: Supplementary file 1 — Supporting File 1: The authors have cited additional references within the Supporting Information [1–27]. [file ANIE-65-e20785-s001.pdf]

Supporting Information

Proton Tautomerism for Anhydrous Superprotonic Conduction in 1,2,3-Triazolium Dihydrogen Phosphate Crystal

Kaito Nishioka, Shun Dekura,* Tomoko Fujino, Motohiro Mizuno, Reiji Kumai, Bo Thomsen, Motoyuki Shiga, Yuta Hori, Yasuteru Shigeta and Hatsumi Mori*

Table of Contents

Experimental Methods S3

Synthesis S3

Preparation of single crystals of 1,2,3-triazolium dihydrogen phosphate (1) S6

Single-crystal X-ray structural analysis S7

Thermal analysis S7

Alternating-current (AC) impedance spectroscopy S7

Fabrication of hydrogen fuel-cell device S8

Solid-state nuclear magnetic resonance (NMR) S9

Theoretical calculations using non-equilibrium molecular dynamics (NEMD) S9

Nudged elastic band calculations S11

***Ab initio* calculation of the barrier of the tautomerism of 1,2,3-$TrzH^+$ in vacuum** S14

Results and Discussion S15

Single crystal structure of 1 ..... S15

Thermal properties S16

Single-crystal anhydrous proton conductivity of 1 ..... S17

Demonstration of fuel-cell device using single crystals of 1 ..... S27

Structural change of 1,2,3-$TrzH^+$ via tautomerism by varying temperature S28

Quantum chemical calculations of a single molecule of 1,2,3-$TrzH^+$ S30

Perturbation energy ($-\mu \cdot \epsilon$) in *ab initio* NEMD S30

***Ab itnitio* NEB calculation of proton tautomerism** S33

References S36

Experimental Methods

Synthesis

General

All manipulations of oxygen- and moisture-sensitive materials were performed using standard Schlenk techniques under an argon atmosphere. Analytical thin-layer chromatography (TLC) was performed on glass plates coated with silica gel (230–400 mesh, 0.25 mm thickness) containing a fluorescent indicator (silica gel $60F_{254}$, Merck).

Apparatus

Flash silica gel column chromatography was performed using a Biotage MPLC system equipped with Biotage® SNAP Ultra packed columns. Proton nuclear magnetic resonance spectra (^{1}H NMR) were recorded on a JEOL JNM AL300 spectrometer (^{1}H NMR: 300 MHz) with solvent resonance as the internal standard (^{1}H NMR, $Si(CH_3)_4$ at 0.00 ppm). ^{1}H NMR data are reported as follows: chemical shift, multiplicity (*s* = singlet, *d* = doublet), *J*-coupling constants (Hz), and relative integrated intensity.

Materials

The following reagents were purchased from commercial suppliers and used without further purification: *N*,*N*-dimethylformamide (DMF, superdehydrated, Wako Pure Chemical Industries), diethyl ether (superdehydrated, Wako Pure Chemical Industries), acetonitrile (MeCN, superdehydrated, Wako Pure Chemical Industries), dichloromethane (superdehydrated, Wako Pure Chemical Industries), acetone (superdehydrated, Wako Pure Chemical Industries), water-d_2 (D_2O, 99.8 atom % D, Wako Pure Chemical Industries), methanol-d_1 (MeOD, 99 atom % D, Cambridge Isotope Laboratories), 1,2,3-triazole (Wako Pure Chemical Industries), phosphoric acid (≥85.0% w/w, Wako Pure Chemical Industries), *n*-butyllithium (*n*-BuLi, 1.56 M in hexane, Kanto Chemical), triisopropylsilylacetylene (Wako Pure Chemical Industries), trimethylsilylazide (Wako Pure Chemical Industries), copper(I) bromide-dimethyl sulfide complex ($CuBr•SMe_2$, Alfa Aesar), tris[(1-benzyl-1*H*-1,2,3-triazol-4-yl)methyl]amine (TBTA, Tokyo Chemical Industry), cesium fluoride (Wako Pure Chemical Industries), phenylacetyl chloride (Wako Pure Chemical Industries), triethylamine (NEt_3, Wako Pure Chemical Industries), acetone (Wako Pure Chemical Industries), dichloromethane (Wako Pure Chemical Industries), ethyl acetate (EtOAc, Wako Pure Chemical Industries), diethyl ether (Wako Pure Chemical Industries), methanol (MeOH, Wako Pure Chemical Industries).

Synthesis of triisopropylsilylacetylene-d_1 (**2-*d*₁**)

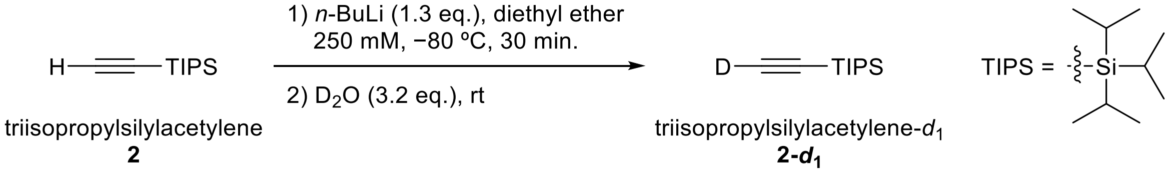

To a solution of triisopropylsilylacetylene (2) (6.20 mL, 27.6 mmol) in diethyl ether (100 mL) was added n-BuLi (23.0 mL, 35.9 mmol, 1.3 eq.) dropwise over 10 min at −80 ºC. The mixture was stirred at the same temperature for 30 min, followed by the addition of D_2O (1.6 mL, 88.4 mmol, 3.2 eq.). The mixture was stirred and allowed to warm to room temperature. After the addition of saturated aqueous NH_4Cl (100 mL), the product was extracted with diethyl ether (100 mL × 3). The combined organic layers were washed with water (100 mL) and brine (100 mL), dried over Na_2SO_4, and concentrated in vacuo. The crude mixture was further dried over molecular sieves 4Afor 24 h and used directly in the next reaction without further purification.

Synthesis of compound **4-*d*₂**

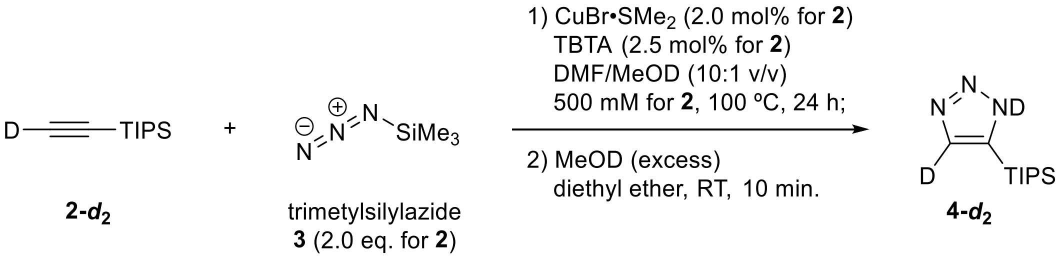

TBTA (366 mg, 0.689 mmol, 2.5 mol% for **2**), CuBr•SMe_2 (114 mg, 0.553 mmol, 2.0 mol% for **2**), and the entire crude mixture containing **2-*d*$_1$** from the previous reaction were dissolved in DMF (50 mL) and MeOD (5.0 mL). Trimethylsilylazide (**3**) (7.25 mL, 55.1 mmol, 2.0 eq. for **2**) was then added, and the mixture was stirred for 24 h at 100 ºC. After the mixture was cooled to room temperature, a 1 M aqueous solution of NH_3 (50 mL) was added, and the mixture was stirred vigorously at room temperature overnight. The bluish-white precipitate was removed by filtration, and the material was extracted with EtOAc (50 mL × 4) from the filtrate. The combined organic layers were washed with 1 M aqueous NH_3 (100 mL), water (100 mL), and brine (100 mL), dried over Na_2SO_4, and concentrated in vacuo. The crude material was purified by flash column chromatography (eluent: 90:10 to 50:50 v/v hexane/EtOAc, column: 50 g × 3) to give a white solid. Finally, the obtained solid was dissolved in a mixed solvent of diethyl ether (10 mL) and MeOD (5 mL) and concentrated in vacuo. This process of dissolving and concentrating was repeated two more times to afford the title compound **4-*d*$_2$** as a white solid (5.18 g, 22.8 mmol) in 82% yield for a two-step transformation from **2**. The deuteration rate of the hydrogen at the carbon atom of **4-*d*$_2$** was determined to be 93.0% based on the intensity of the peak for the signal (0.07H) in ^{1}H NMR spectrum (Figure S1). ^{1}H NMR (300 MHz, RT, $CDCl_3$) δ 7.82 (*s*, 0.07H), 1.36 (sextet, J = 7.3 Hz, 3H), 1.11 (*d*, J = 7.3 Hz, 18H).

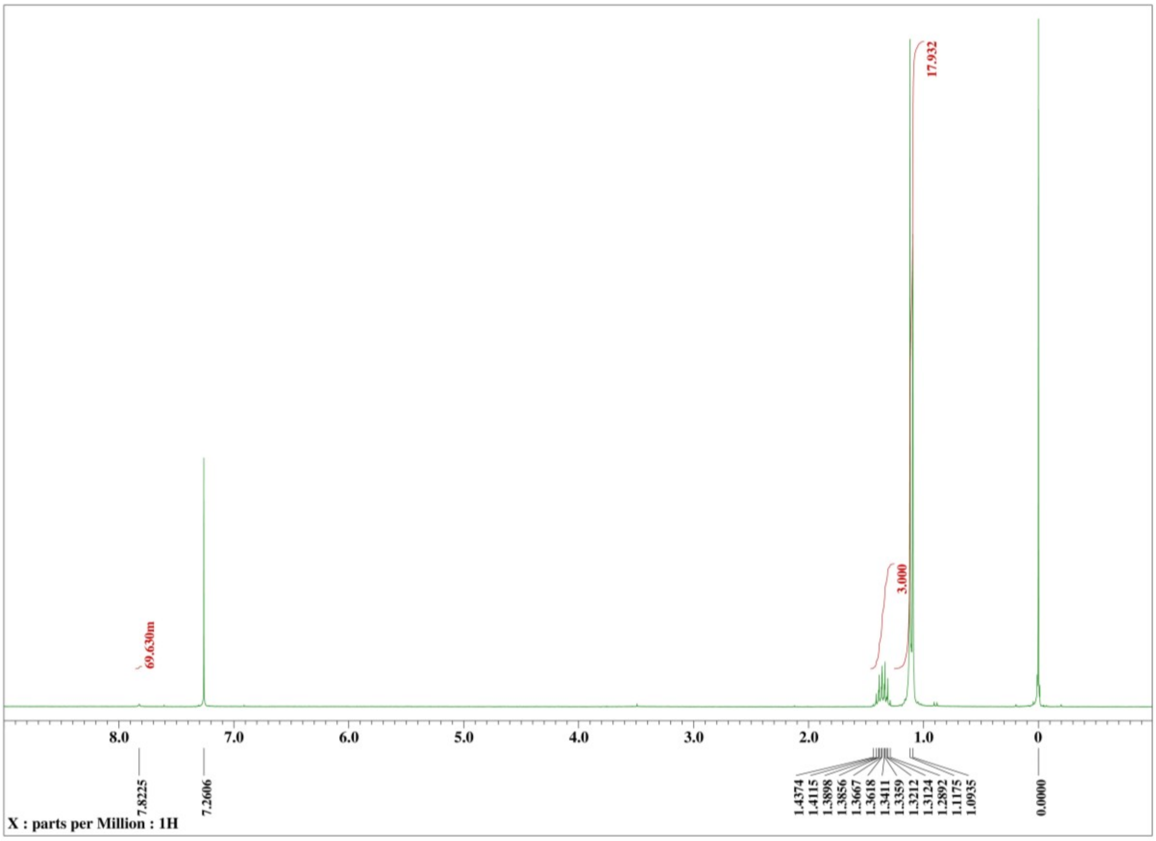

Figure S1. Proton NMR spectrum of 4-*d*₂ (300 MHz, RT, CDCl₃, relaxation delay: 30 s).

Synthesis of 1,2,3-triazolium-d_2 dihydrogen phosphate (**1-*d*₂**)

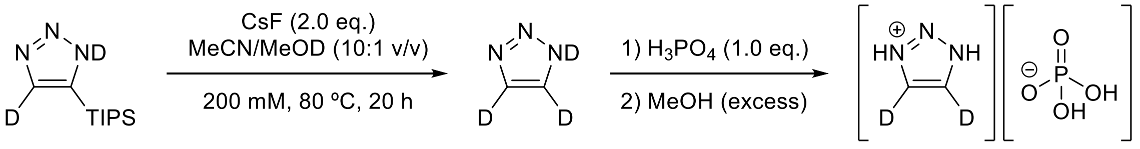

4-*d*₂ 1,2,3-triazole-d_3 1,2,3-triazolium-d_2 dihydrogen phosphate **1-*d*₂**

Cesium fluoride (4.00 g, 26.3 mmol, 2.0 eq.) was dried with a heat gun under reduced pressure. **4-*d*₂** (3.00 g, 13.2 mmol), MeOD (6.0 mL), and acetonitrile (60 mL) were then added, and the suspension was stirred at 80 ºC for 20 h. The reaction mixture was quenched with NH_4Cl (1.00 g) and MeOH (50 mL) and concentrated in vacuo. Insoluble materials were removed by filtration through a pad of silica gel (eluent: EtOAc) to obtain crude oil containing 1,2,3-triazole-d_3. The crude product was treated with H_3PO_4 (0.90 mL, 13.2 mmol, 1.0 eq.), followed by the addition of MeOH (40 mL), and concentrated in vacuo. The resulting solid was dissolved in MeOH (40 mL), and volatile materials were removed in vacuo to promote D/H substitution at the N^1-position. This dissolution–evaporation process was repeated once more, after which the residue was sonicated in $CHCl_3$ (40 mL), filtered, and dried in vacuo at 60 ºC for 1 h to yield **1-*d*₂** as a white powder in 84% yield (1.88 g, 11.1 mmol) over a two-step transformation from **4-*d*₂**.

Determination of the deuteration rate of **1-*d*₂**

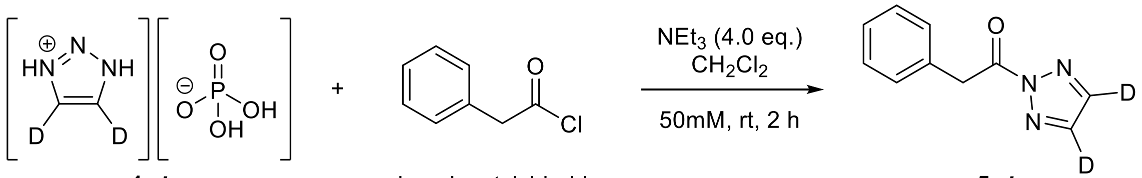

Phenylacetylchloride (310 µL, 2.35 mmol, 4.0 eq.) was added to a solution of **1-*d*$_2$** (100 mg, 0.592 mmol), triethylamine (330 µL, 2.37 mmol, 4.0 eq.), and CH_2Cl_2 (12 mL). The mixture was stirred at room temperature for 2 h, then quenched with a saturated aqueous $NaHCO_3$ solution (10 mL). The organic layer was washed with saturated aqueous $NaHCO_3$ (10 mL × 2), water (10 mL), and brine (10 mL), dried over Na_2SO_4, and concentrated in vacuo. The crude product was purified by flash column chromatography (eluent: 80:20 to 60:40 v/v hexane/EtOAc, column: 25 g) to give ***5-d*$_2$** as a white solid in 60% yield (66.8 mg). The deuteration rate was determined to be 92.1% based on the integrated intensity of the corresponding proton in the ^{1}H NMR spectrum (Figure S2), consistent with reported values.[1]

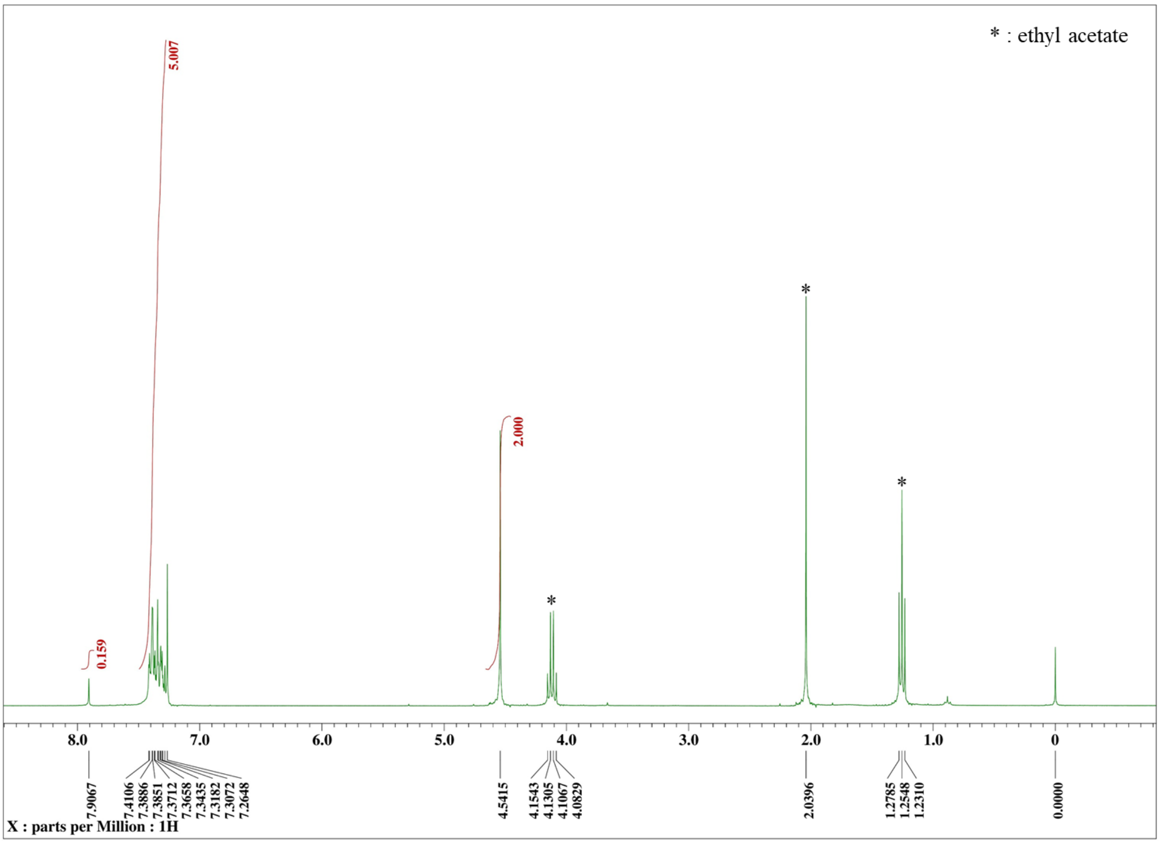

Figure S2. Proton NMR spectrum of 5-*d*2 (300 MHz, RT, $CDCl_3$, relaxation delay: 30 s).

Preparation of single crystals of 1,2,3-triazolium dihydrogen phosphate (1)

Phosphoric acid (3.45 mL, 50.4 mmol) was added to a solution of 1,2,3-triazole (2.90 mL, 50.0 mmol)

in acetone (50 mL) to form a white precipitate. The suspension was stirred for 10 min at room temperature and concentrated in vacuo to yield white crude crystals of 1,2,3-triazolium dihydrogen phosphate (**1**) (8.54 g). Single crystals of **1** were obtained via the vapor diffusion method. A solution of the crude crystals of **1** (1.00 g) in acetone (300 mL) was slowly exposed to dichloromethane vapor, yielding plate-shaped colorless single crystals of **1**. The structural integrity was confirmed via single-crystal structural analysis (Table S1 and Figure S6).

Single-crystal X-ray structural analysis

X-ray diffraction (XRD) measurements for **1** were performed on a Mercury II CCD X-ray diffractometer (Rigaku Corp., Japan) (Mo K_{α}, λ = 0.71073 Å). XRD measurements for **1-*d*₂** were performed on a XtaLab P200 X-ray diffractometer (Rigaku Corp., Japan) (Mo K_{α}, λ = 0.71073 Å). The initial structures were determined using direct methods (SHELXT version 2018/2) and refined using a full-matrix least squares technique (SHELXL version 2018/3) with Olex2-1.5 (OlexSys).[2] Anisotropic thermal parameters were applied to the non-hydrogen atoms.

Thermal analysis

The melting point of **1** was determined using an MPA-100 Automated Melting Point System (Stanford Research Systems, Inc.). The single crystals were loaded into a glass capillary, which was inserted into the instrument heating slot and heated from 313 to 433 K at a rate of 1 K min^{-1}. The thermal behavior of the crystals of **1** was evaluated via differential scanning calorimetry (DSC) using a Netzsch DSC 200 F3-T21 Maia calorimeter with an Al reference. The single crystals (6.2552 mg) were encapsulated in an Al pan and heated from 293 to 423 K at a rate of 5 K min^{-1} under N_2 gas flow. The thermal stability of the crystals of **1** was evaluated via thermogravimetry/differential thermal analysis (TG/DTA) using a SII EXSTAR TG/DTA7300 with an Al reference. The single crystals (3.2696 mg) were placed in an Al pan and heated from 303 to 823 K at a rate of 1 K min^{-1} under N_2 gas flow.

Alternating-current (AC) impedance spectroscopy

AC impedance spectroscopy measurements were conducted using the quasi-four-probe method with an Agilent Impedance Analyzer 4294A and a homemade impedance probe. As-grown single crystals of **1** were cut into blocks (typical size: 0.2 × 0.2 × 0.2 mm^3) to expose the desired facets, the Miller indices of which were confirmed in advance via XRD, for the anisotropy measurements. The electrodes were separately attached to the two opposite sides of a crystal by using Ag paste (DOTITE D-500, Fujikura Kasei Co., Ltd.) and Au wires (φ: 10 μm), and the crystal was covered with ThreeBond epoxy resin 2088E. The complex impedance of the sample was measured from 40 Hz to 5 MHz at intervals of 5.0 K from 315.5 to 395.5 K; the temperature was controlled using a Lakeshore Temperature Controller 335 equipped with a 50-Ω heater.

The complex impedance Z was fitted by the following formula,

$$Z = \frac{R_\mathrm{S}}{1 + (i\omega)^p T_{\mathrm{CPE_S}} R_\mathrm{S}} + R_\mathrm{C},$$

where i is the imaginary unit, ω is an angular frequency, R_S is a sample resistance, $T_{\mathrm{CPE_S}}$ is a constant-phase-element coefficient, p is a constant-phase-element exponent, and R_C is a contact resistance. When the Z consists of two components, the fitting function for Z becomes

$$Z = \frac{R_\mathrm{S1}}{1 + (i\omega)^{p1} T_{\mathrm{CPE_{S1}}} R_\mathrm{S1}} + \frac{R_\mathrm{S2}}{1 + (i\omega)^{p2} T_{\mathrm{CPE_{S2}}} R_\mathrm{S2}} + R_\mathrm{C}.$$

The proton conductivity σ and the relative permittivity $\varepsilon/\varepsilon_0$ were calculated as follows,

$$\sigma = \frac{d}{R_\mathrm{S1} S}$$

$$\varepsilon/\varepsilon_0 = \frac{T_{\mathrm{CPE_S}}^{\frac{1}{p}} R_\mathrm{S}^{1-\frac{1}{p}} d}{\varepsilon_0 S},$$

where d is the distance between the electrodes, S is the area of the electrodes, and ε_0 is the permittivity of the vacuum.

The E_a values were estimated using the Arrhenius equation.

$$\sigma T = \sigma_0 \exp\left(-\frac{E_\mathrm{a}}{k_\mathrm{B} T}\right),$$

where σ_0 is a prefactor and k_B is the Boltzmann constant.

Fabrication of hydrogen fuel-cell device

The catalyst electrodes were separately attached to the two opposite sides of as-grown single crystals of **1** (typical size: 0.5 × 2 × 3 mm^3) using Pt/C paste (FC-R&D Co., Ltd.) and Au wires (φ: 15 μm) (Figures S3a and 3b). The crystal was placed on a hole (φ: 2 mm) made on the surface of the polystyrene case (1 × 3 × 3 cm^3) and sealed with epoxy resin such that one electrode surface was exposed to the internal atmosphere of the case and the other was exposed to the external atmosphere. The attached Au wires were connected via Cu wire (φ: 0.2 mm) and soldered to a digital clock as an output device. During operation, H_2 gas generated by electrolysis of water was sent continuously into the styrene case, and O_2 in the air was used (Figure S3c).

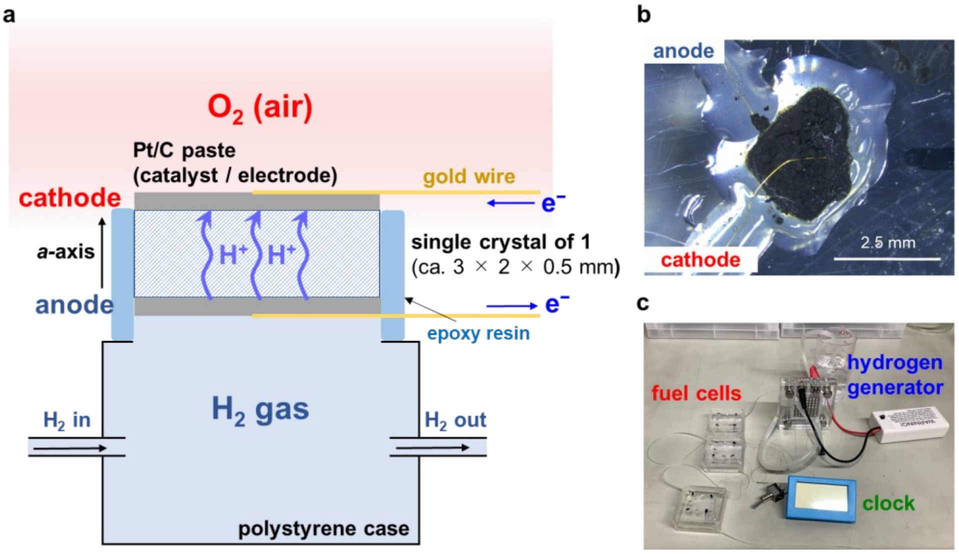

Figure S3. Hydrogen fuel-cell device. a, Schematic diagram of the fabricated fuel cell. **b**, Single crystal of **1** used in the fuel cell, attached with electrodes and gold wires. **c**, Photograph of the fuel cell in operation.

Solid-state nuclear magnetic resonance (NMR)

Solid-state ^{2}H NMR spectra were measured using a Bruker Avance III 400 MHz spectrometer with a ^{2}H resonance frequency of 61.431 MHz. Single crystals of **1-*d*₂** were crushed to prepare a powder sample, which was sealed in a glass tube. The ^{2}H NMR wide-line spectra were measured using a solid echo sequence $(90°)_x–\tau–(90°)_y–\tau–t_{acq}$, where τ and t_{acq} are the echo interval and acquisition time, respectively. The 90° pulse width, τ, and t_{acq} were 2.85, 20, and 450 µs, respectively. The temperature was controlled using a dry N_2 gas flow. Simulations of the wide-line ^{2}H NMR spectra were performed using homemade Fortran programs with double precision.[3] The spectral simulation was conducted using the three-site jump model of deuterons describing the libration motion of the **1,2,3-Trz-d_2** molecules (Figure S4).

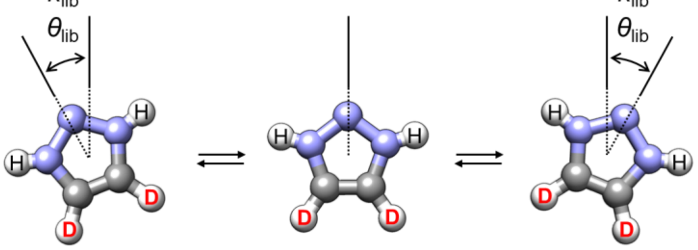

Figure S4. Schematic image of the three-site jump model adopted for the spectral simulations. Libration motion of **1,2,3-TrzH^{+}-d_2** molecule is assumed with libration angle θ_{lib} and the frequency k_{lib}.

Theoretical calculations using non-equilibrium molecular dynamics (NEMD)

Non-equilibrium molecular dynamics(NEMD)

The NEMD simulation was conducted using the color-diffusion method. We consider a system of N atoms contained in a periodic box, in which an external electrostatic field $\boldsymbol{\epsilon}$ is applied. The total Hamiltonian is expressed as

$$H = H_{\text{sys}} + H'$$

The first term, H_{sys}, is the Hamiltonian of the atomic system expressed as

$$H_{\text{sys}} = \sum_{i=1}^{N} \frac{\mathbf{p}_i^{\,2}}{2m_i} + V_{\text{BO}}(\mathbf{r}_1, \cdots, \mathbf{r}_N),$$

where $\mathbf{p}_i$ and m_i are the momentum and mass of atom i, respectively, and V_{BO} is the Born–Oppenheimer potential obtained from DFT calculations. The second term describes the perturbation due to dipole coupling

$$H'(t) = -\boldsymbol{\mu}(t) \cdot \boldsymbol{\epsilon},$$

where the dipole moment of the system at time t is expressed as

$$\boldsymbol{\mu}(t) = \sum_{i=1}^{N} q_i \mathbf{r}_i.$$

Here, $\mathbf{r}_i$ is the position at time t (unwrapped with respect to the periodic boundary condition), and q_i is the charge of atom i.

By solving the canonical equations of motion,

$$\frac{d\mathbf{r}_i}{dt} = \frac{\mathbf{p}_i}{m_i}, \frac{d\mathbf{p}_i}{dt} = -\frac{\partial V_{\text{BO}}}{\partial \mathbf{r}_i},$$

the charge flux of the system is given as

$$\mathbf{J}(t) = \frac{d\boldsymbol{\mu}(t)}{dt} = \frac{d}{dt}\sum_i q_i \mathbf{r}_i = \sum_i q_i \dot{\mathbf{r}}_i,$$

which can be collected from the NEMD trajectory. Assuming the linear response with respect to ϵ, the conductivity can be obtained as

$$\sigma = \lim_{\epsilon \to 0} \frac{\langle J(t) \rangle_\epsilon}{\epsilon V} \simeq \frac{\langle J(t) \rangle_\epsilon}{\epsilon V} = \frac{1}{\epsilon V}\frac{d\langle \mu(t) \rangle_\epsilon}{dt} = -\frac{1}{\epsilon^2 V}\frac{d\langle H'(t) \rangle_\epsilon}{dt},$$

where $J(t)$ is the component of $\mathbf{J}(t)$ along the direction of $\boldsymbol{\epsilon}$, and ϵ is the norm of $\boldsymbol{\epsilon}$, and V is the volume of the system.

The *ab initio* NEMD simulations were conducted using the path integral molecular dynamics (PIMD) software package.[4] The electronic structure calculations, used to evaluate potential energies and gradients, were conducted on the fly using the CP2K quantum chemistry program,[5] which was linked to the PIMD code as a library.

The revised Perdew–Burke–Ernzerhof[6] (RPBE) DFT functional was employed using the LIBCX library.[7,8] A triple-zeta valence plus polarization (TZV2P) basis set[9] associated with Goedecker-Teter-Hutter (GTH) pseudopotentials[10] was used in combination with a plane-wave basis set with a cutoff of 500 Ry. Grimme's D3 dispersion correction[11,12] was employed to account for van der Waals interactions. The ELPA[13] and FFTW[14] libraries were used to accelerate the solution of the electronic-structure eigenvalue equations.

NEMD simulations were performed for the neutral, positively charged, and negatively charged systems.

The neutral system consisted of four phosphate and triazole units contained in a periodic unit cell, while the positively and negatively charged systems had a proton added or removed, respectively. The cell parameters were adopted from the experimental structure at 298 K. A uniform background charge was applied for the charged systems. The timestep was set to Δt = 0.25 fs, and the simulations were conducted for 1,000,000 steps each, with atomic velocities initialized from the Maxwell–Boltzmann distribution at 390 K.

The color-diffusion algorithm[15–17] was used with an applied electrostatic field of ϵ = 1.0×10^9 V/m along the *a*, *b*, and *c* axes. Under this field, the system maintained a steady state, and the dipole changed gradually over the NEMD simulation time of 250 ps. The simulations were thus performed on nine systems in total (neutral, positive, and negative systems, each with the electrostatic fields applied along the *a*, *b*, and *c* axes).

Although the applied field does not directly affect the electronic structure calculations, it introduces a gradient based on the color charges assigned to each atom type in the NEMD simulation to drive proton transfer. Atomic charges for the interaction between atom types and the applied field were assigned based on averaged Mulliken charges (q) obtained from the neutral crystal structure: q_{P} = 0.603791667, q_{O} =0.698375, q_{H} =0.552666667, q_{N} =-0.227833333, and q_{C} =-0.23025. Further details on the color-diffusion algorithm and the derivation of σ from the slope of the regression between simulation time and perturbation strength are provided above.

Nudged elastic band calculations

Solid-state DFT calculations were performed using OpenMX software (Ver. 3.9) based on optimized localized basis functions and pseudopotentials (PPs). The basis functions used were H6.0-s2p1, C6.0-s2p2d1, N6.0-s2p2d1, O6.0-s2p2d1, and P7.0-s2p2d1f1 for H, C, N, O, and P, respectively. The abbreviations were established as follows, taking C6.0-s2p2d1 as a representative example: C is the atomic symbol, 6.0 represents the cutoff (Bohr) radius of the confinement-scheme generation, and s2p2d1 indicates the employment of two, two, and one optimized radial functions for the s-, p-, and d-orbitals, respectively. The radial functions were optimized using a variational optimization method.[18,19] As valence electrons in the PPs, 1s for H; 2s and 2p for C, N, and O; and 3s and 3p for P were included. All PPs and pseudo-atomic orbitals used in the study were taken from the OpenMX website database (2019);[20] these were benchmarked using the delta gauge method.[21] Real-space grid techniques were used for the numerical integrations and for the Poisson-equation solution using FFT; an energy cutoff of 300 Ryd was employed.[22] The generalized gradient approximation proposed by Perdew, Burke, and Ernzerhof was used for the exchange–correlation functional.[23] For all systems considered in this study, an electronic temperature of 300 K was employed to count the number of electrons using the Fermi–Dirac function. For the k-point sampling, a regular mesh of 2 × 2 × 2 was used.

Structural optimizations, for which the lattice parameters were fixed and only the atomic coordinates were optimized, were performed for each of the structures with protons on different proton-accepting sites (Figure S5). The unit cell, which contains four **1,2,3-TrzH$^+$** and four $H_2PO_4^-$, was used as the computational

cell without symmetry constraints. The cell parameters and the initial structure for the perfect-crystal system were adopted from the experimentally obtained X-ray structure at 298 K. For the proton-excess crystal system, the initial structure comprised one proton per unit cell added to the position on the N^2 of the triazole in the crystal structure with an entire charge of +1. For the proton-deficient crystal system, a structure was employed in which one proton per unit cell was removed from the N^3 position of the triazole in the crystal structure, resulting in a net charge of −1. The other structural models for the metastable states were adopted so that the focused proton in the unit cell could migrate through the crystal to return to the original crystallographic position. The minimum energy paths and the corresponding E_a values were calculated using the NEB method[24] with the input of the two optimized structures obtained in this study as the initial and final states, respectively.

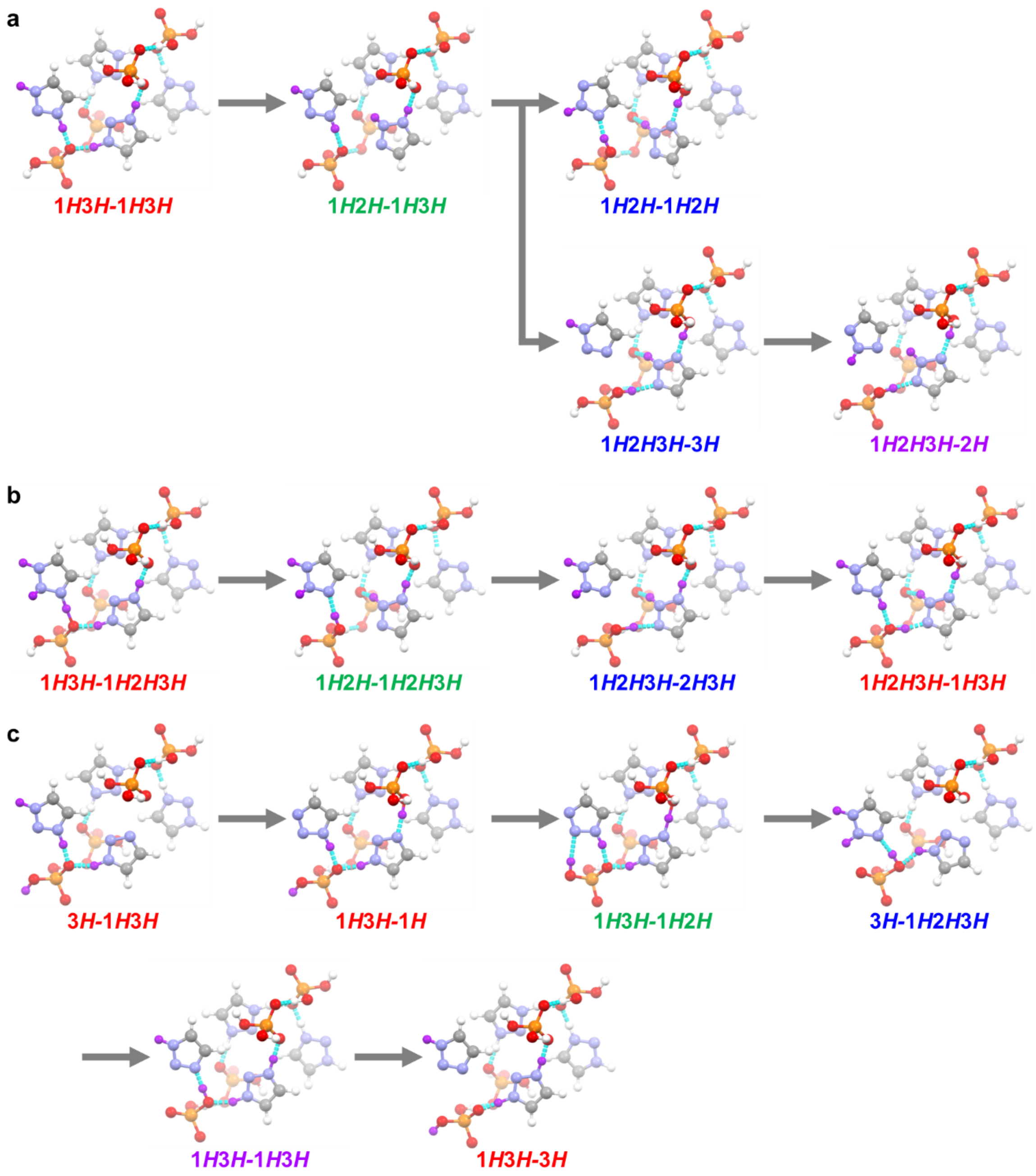

Figure S5. Structures before optimization in NEB calculations. Structures of local minimum states before optimization **a**, in the perfect crystal, **b**, in the proton-doped crystal, and **c**, in the defect-doped crystal (gray: C, white: H, blue: N, red: O, orange: P; conducting protons are colored purple). Structural optimizations were performed using each structure as input.

Ab initio calculation of the barrier of the tautomerism of 1,2,3-TrzH$^+$ in vacuum

Quantum chemical calculations were performed using Gaussian 16 software (Rev. C.01).[25] Geometry optimizations of **1*H*,3*H*-TrzH$^+$**, **2*H*,3*H*-TrzH$^+$**, and the transition state between them were performed using the M062X DFT functional[26] and the 6-31++G(d,p) basis set. Vibrational frequency calculations were performed at the same level of theory used for the optimized geometries. All stationary points were identified as stable minima by confirming that all vibrational frequencies were real and positive. The transition state was characterized by the presence of a single imaginary frequency. To obtain the potential energy curve between **1*H*,3*H*-TrzH$^+$** and **2*H*,3*H*-TrzH$^+$**, intrinsic reaction coordinate (IRC) calculations[27] were performed using the same computational setup. The activation energy for proton tautomerism of a single molecule of **1,2,3-TrzH$^+$** is calculated as the energy difference between **1*H*,3*H*-TrzH$^+$** and the transition state.

Results and Discussion

Single crystal structure of 1

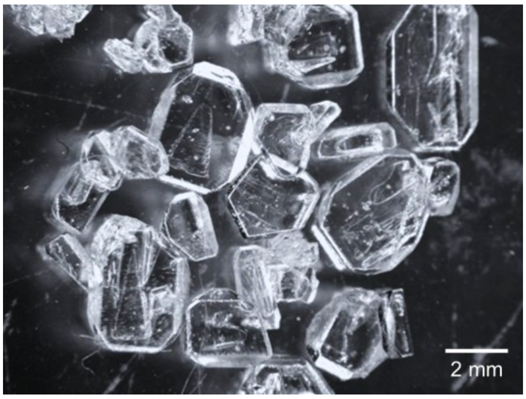

Figure S6. Photograph of the single crystals of 1.

Table S1 Crystallographic data of 1 and 1-*d*₂

	**1**	**1-*d*₂**
Temperature / K	298	298
Empirical formula	$C_2H_6N_3O_4P$	$C_2H_4D_2N_3O_4P$
Crystal system	Monoclinic	Monoclinic
Space group	$P2_1/c$	$P2_1/c$
a / Å	9.2759(5)	9.2629(3)
b / Å	9.7820(6)	9.7612(3)
c / Å	7.1311(5)	7.1220(2)
β / °	92.387(5)	92.405(3)
Volume / $Å^3$	646.49(7)	643.38(3)
Z value	4	4
Z' value	1	1
R_1 (I >= 2.00σ(I))	0.0460	0.0293
wR_2 factor (all)	0.1359	0.0863
Goodness of fit	1.100	1.078
CCDC	2471904	2471921

Thermal properties

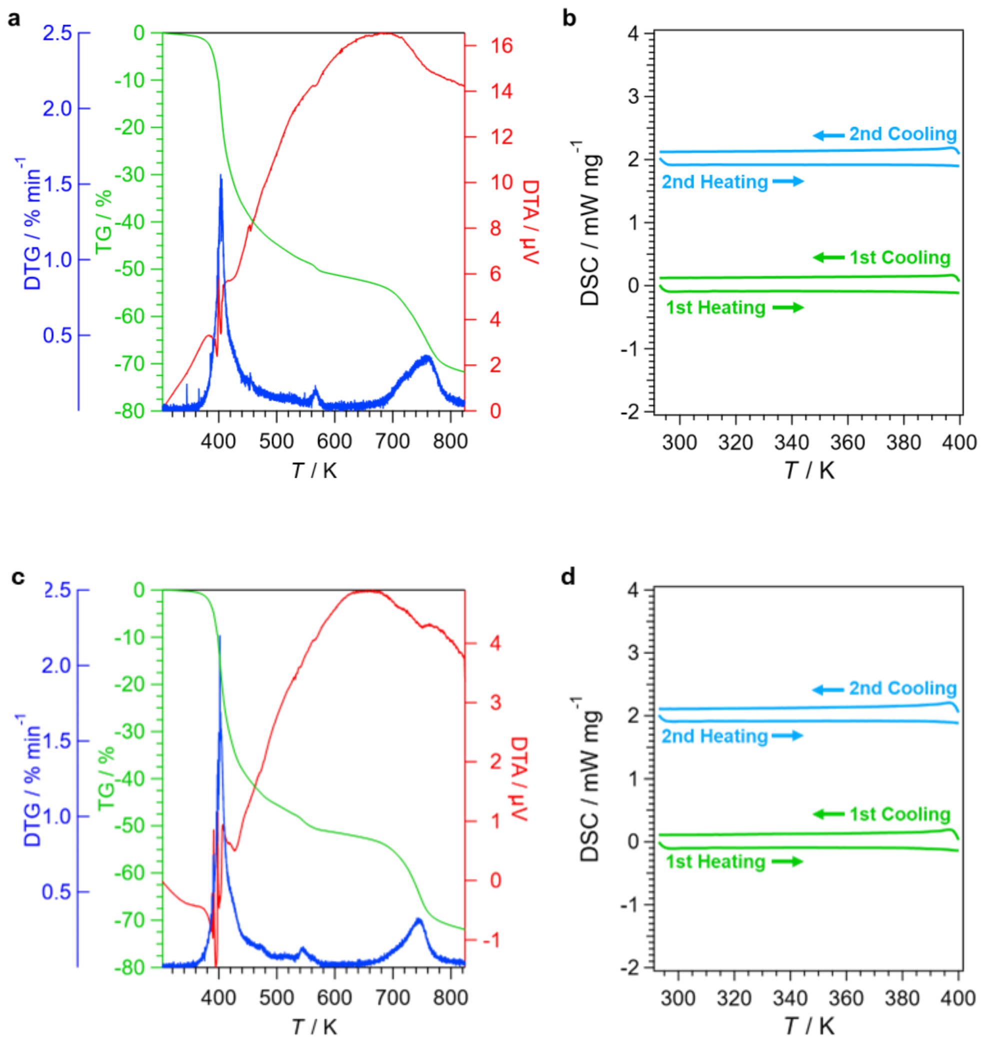

Figure S7. TG/DTA and DSC of a,b 1 and c,d 1-*d*$_2$. a, c From 303 to 823 K at 1 K min^{-1} under nitrogen flow. **b, d** From 293 to 400 K at 5 K min^{-1} under nitrogen flow. Heating and cooling were repeated for two cycles. The DSC curve in the second cycle is offset for clarity.

Single-crystal anhydrous proton conductivity of 1

AC impedance spectra of the bare single crystal of **1** were measured along the ~~*c*~~*a* axis (Figure S8a). However, the single crystal appeared to fluidize above 360.5 K, which is significantly lower than the actual melting point of 413 K~~.~~, leading to a dramatic enhancement in conductivity. The impedance spectra were fitted with two-component equivalent circuits containing R_{S1}/CPE_{S1} and R_{S2}/CPE_{S2} (Figure S8c). R_{S1}/CPE_{S1} appeared from 315.5 K to 370.5 K, and R_{S2}/CPE_{S2} did from 360.5 K to 380.5 K. The relative permittivity of R_{S1}/CPE_{S1} and R_{S2}/CPE_{S2} was calculated by fitting of the impedance spectra (Figure S8b). While the relative permittivity of R_{S1}/CPE_{S1} showed almost no variation with temperature and remained on the order of 10^1, that of R_{S2}/CPE_{S2} increased sharply with rising temperature, reaching values exceeding 10^7 at its maximum. Although the single crystal of **1** is non-dielectric, its constituent molecules are charged; therefore, the sharp increase in the relative permittivity is considered to be caused by fluidization of the sample. This behavior may result from instability during long-term measurements at elevated temperature. To investigate long-term thermal stability below the melting point, we monitored the weight loss of the sample kept at 349 K. A weight loss of approximately 3% was observed after 12 h (Figure S8k), suggesting that the melting-like behavior observed in the AC impedance measurement was due to evaporation of **1,2,3-Trz** from the sample. To prevent evaporation of **1,2,3-Trz** and achieve stable measurements near the actual melting point of **1**, the conductivity was measured using the single crystal covered with resin (Figure S9).

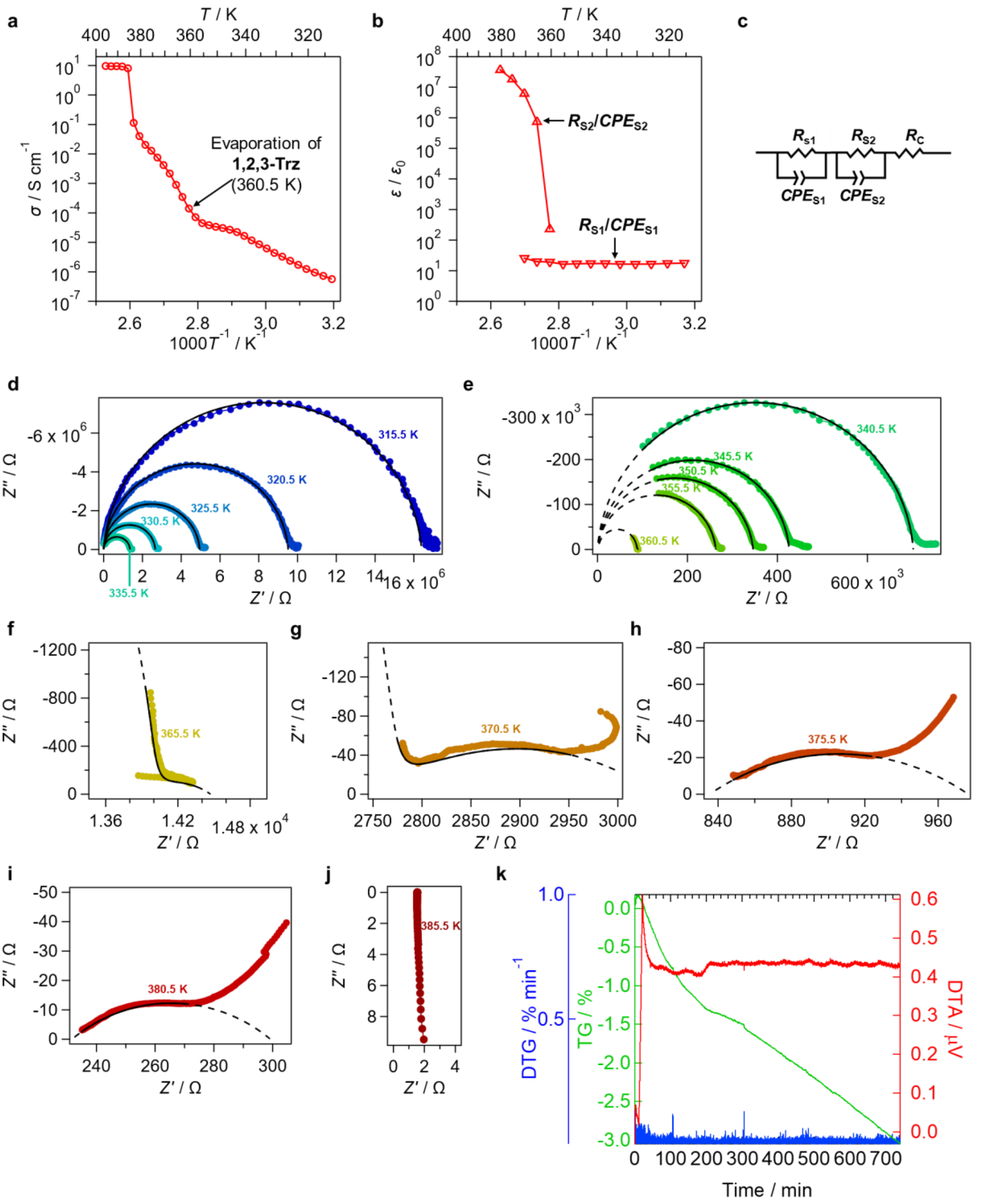

Figure S8. Evaporation of 1,2,3-Trz. a, Anhydrous proton conductivity of the bare single crystal of **1** along the *a*-axis. The conductivity at 40.00 Hz obtained from AC impedance spectra is plotted. **b**, Relative permittivity calculated by fitting the spectra with two-component equivalent circuit **c**, Equivalent circuit used for fitting analyses (R_{S1}: sample resistance 1, R_{S2}: sample resistance 2, R_C: contact resistance, CPE_{S1}: constant phase element 1 for the sample, CPE_{S2}: constant phase element 2 for the sample,). **d–j**, Cole–Cole plots at different temperatures. **k**, Temporal change in the weight of single crystals of **1** heated at 349 K under nitrogen flow.

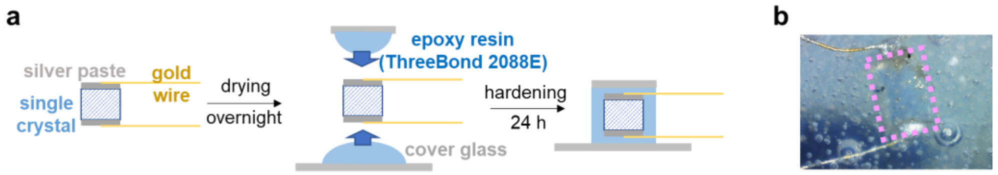

Figure S9. Prevention of evaporation of 1,2,3-triazole. a, Preparation of covered single crystals of **1** for AC impedance measurements. **b**, The single crystal of **1** embedded in resin. The pink dotted line indicates the shape of the crystal.

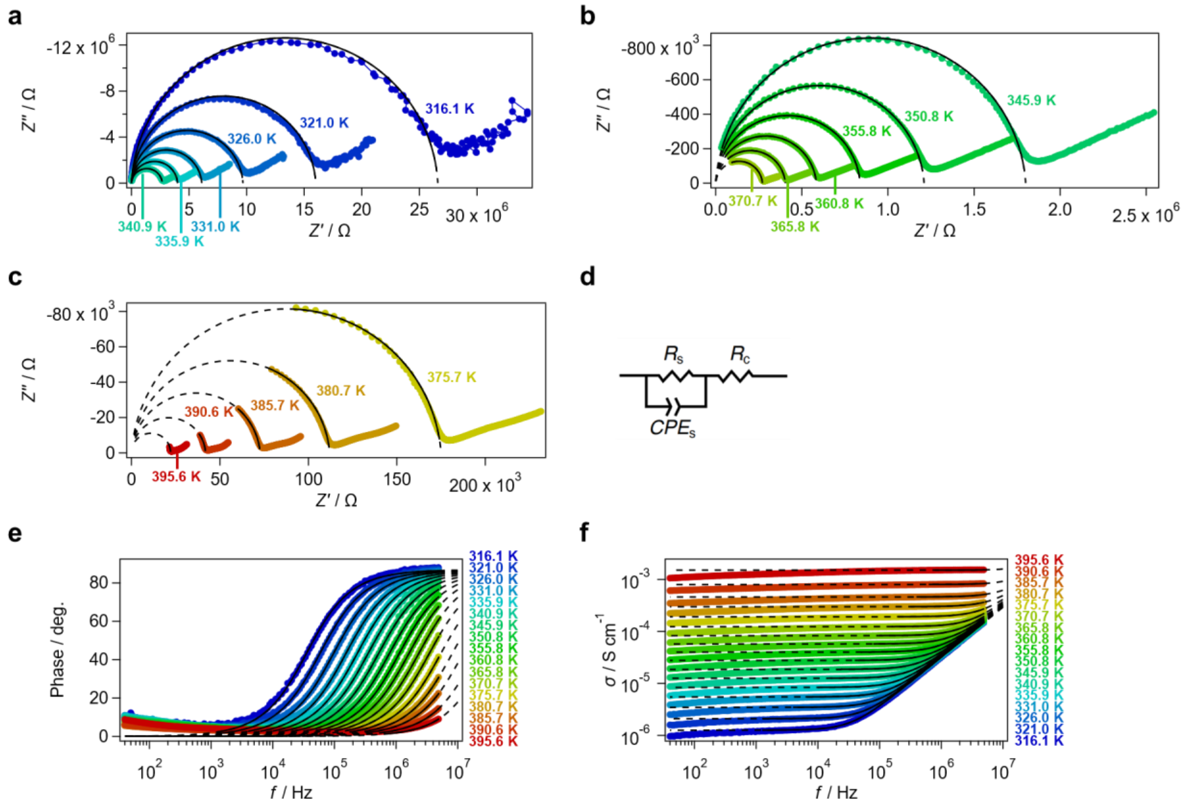

Figure S10. Frequency-dependent AC impedance data for the single crystal of 1 measured along the *a*-axis. a–c, Cole–Cole plots at different temperatures. **d**, Equivalent circuit used for fitting analyses (Rs: sample resistance, Rc: contact resistance, *CPE*s: constant phase element for the sample). **e**, **f**, Bode plots at different temperatures. Colored circles: experimental data, solid curves: fitting curves assuming the equivalent circuit shown in **d**, dashed curves: extrapolated curves assuming the same equivalent circuit.

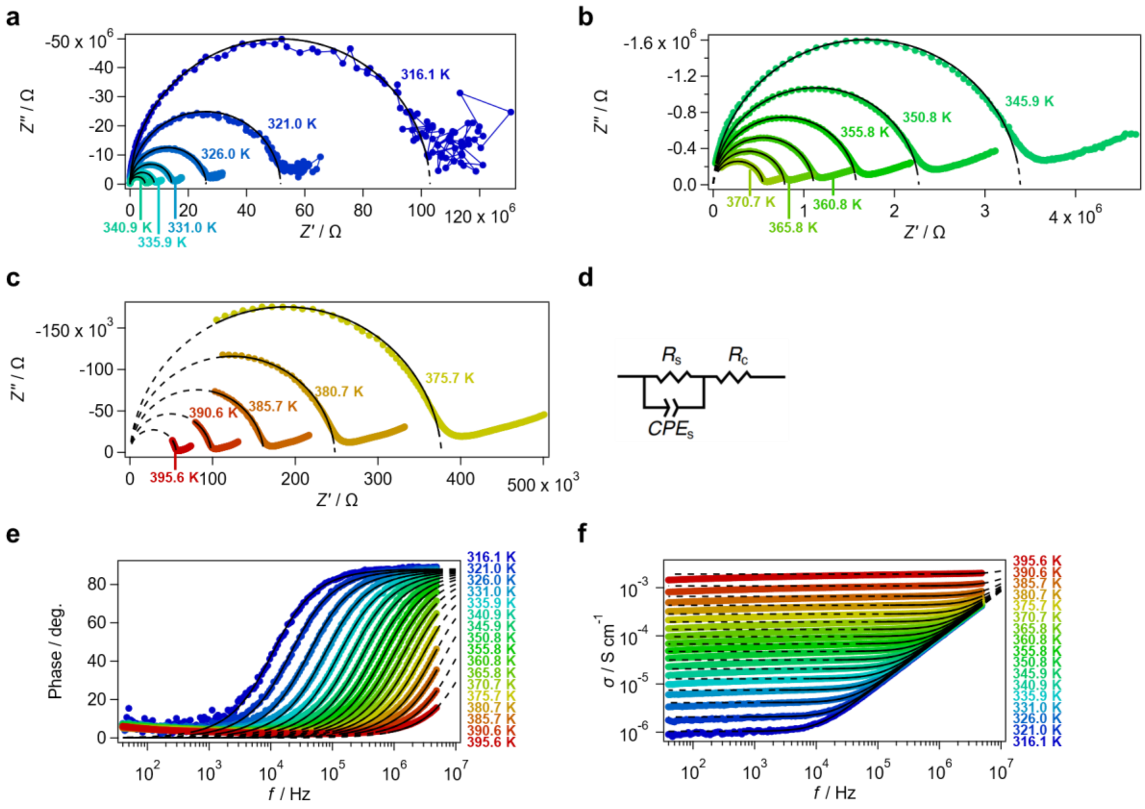

Figure S11. Frequency-dependent AC impedance data for the single crystal of 1 measured along the *b*-axis. a–c, Cole–Cole plots at different temperatures. **d**, Equivalent circuit used for fitting analyses (*R*s: sample resistance, *R*c: contact resistance, *CPE*s: constant phase element for the sample). **e**, **f**, Bode plots at different temperatures. Colored circles: experimental data, solid curves: fitting curves assuming the equivalent circuit shown in **d**; dashed curves: extrapolated curves assuming the same equivalent circuit.

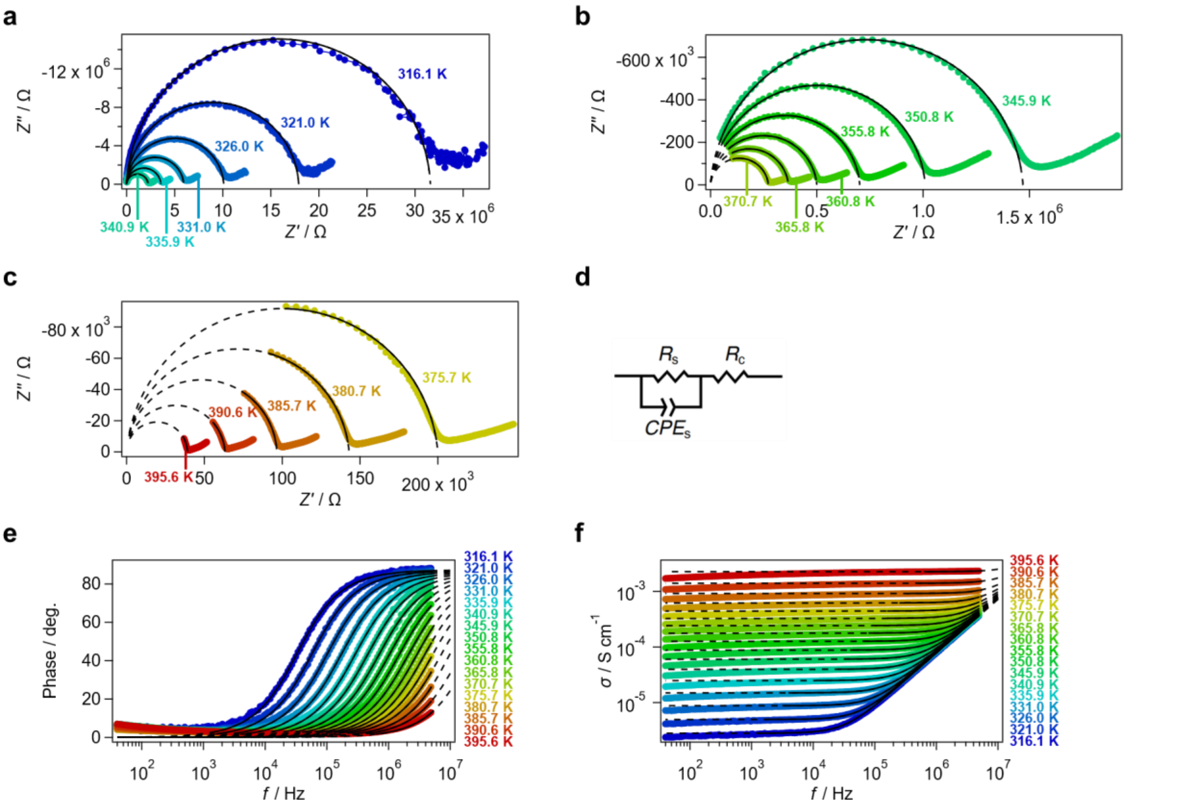

Figure S12. Frequency-dependent AC impedance data for the single crystal of 1 measured along the *c*-axis. a–**c**, Cole–Cole plots at different temperatures. **d**, Equivalent circuit used for fitting analyses (*R*s: sample resistance, *R*c: contact resistance, *CPE*s: constant phase element for the sample). **e**, **f**, Bode plots at different temperatures. Colored circles: experimental data, solid curves: fitting curves assuming the equivalent circuit shown in **d**, dashed curves: extrapolated curves assuming the same equivalent circuit.

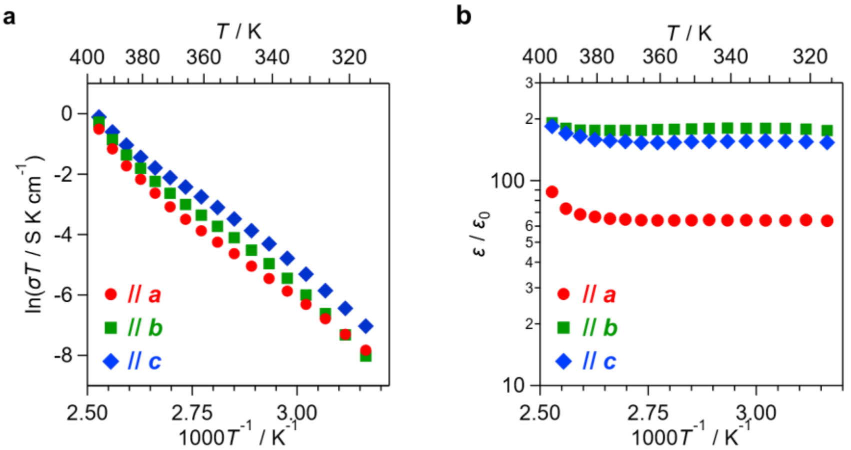

Figure S13. Arrhenius plots of the anhydrous proton conductivity and temperature dependence of relative permittivity of 1. a, Arrhenius plots. **b**, Relative permittivity. Red circles: along the *a*-axis, green squares: along the *b*-axis, blue diamonds: along the *c*-axis.

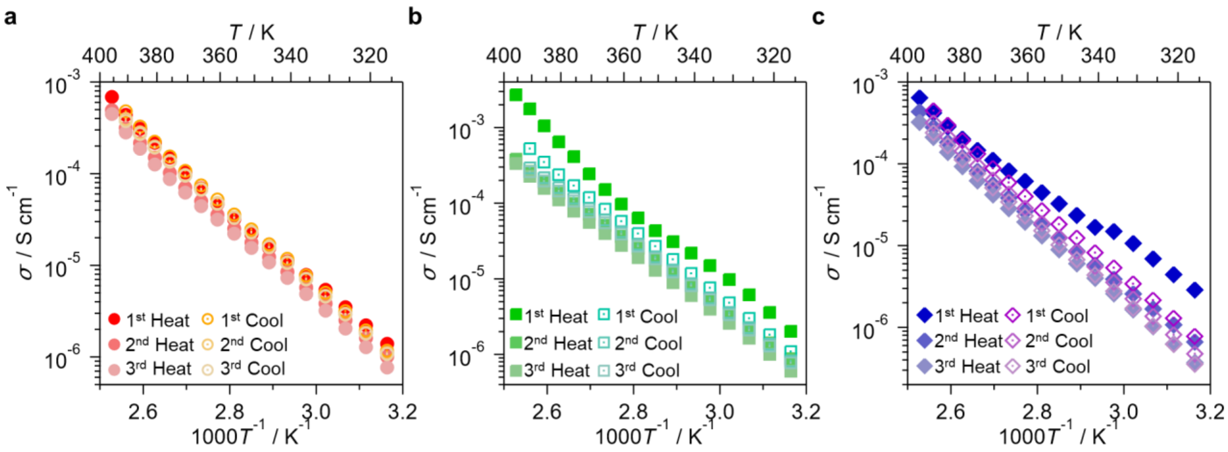

Figure S14. Three-cycle temperature dependence of anhydrous proton conductivity (σ) along a, the *a*-axis, b, the *b*-axis, and c, the *c*-axis. Closed circles: 1st, 2nd, and 3rd heating conditions along the *a*-axis, open circles: 1st, 2nd, and 3rd cooling conditions along the *a*-axis, closed square: 1st, 2nd, and 3rd heating conditions along the *b*-axis, open square: 1st, 2nd, and 3rd cooling conditions along the *b*-axis, closed diamond: 1st, 2nd, and 3rd heating conditions along the *c*-axis, open diamond: 1st, 2nd, and 3rd cooling conditions along the *c*-axis.

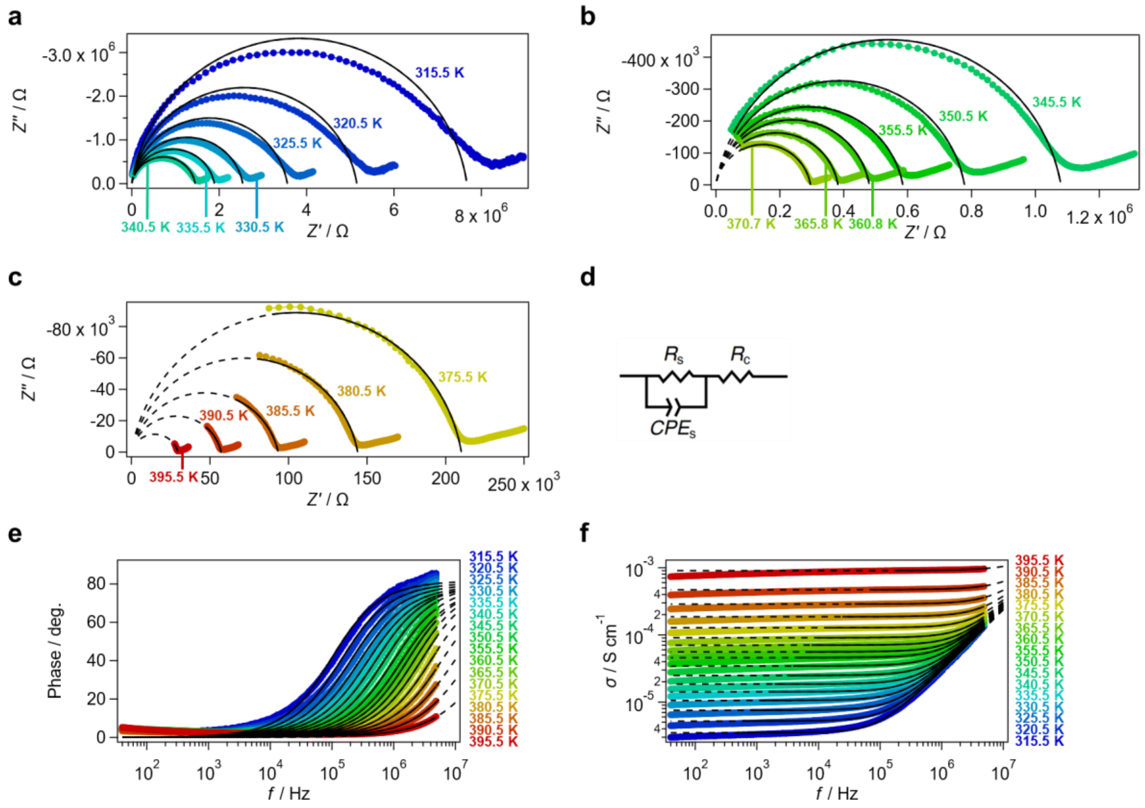

Figure S15. Frequency-dependent AC impedance data for the single crystal of 1-d_2 measured along the *a*-axis. a−c, Cole–Cole plots at different temperatures. **d**, Equivalent circuit used for fittinganalyses (*R*s: sample resistance, *R*c: contact resistance, *CPE*s: constant phase element for the sample). **e**, **f**, Bode plots at different temperatures. Colored circles: experimental data, solid curves: fitting curves assuming the equivalent circuit shown in **d**, dashed curves: extrapolated curves assuming the same equivalent circuit.

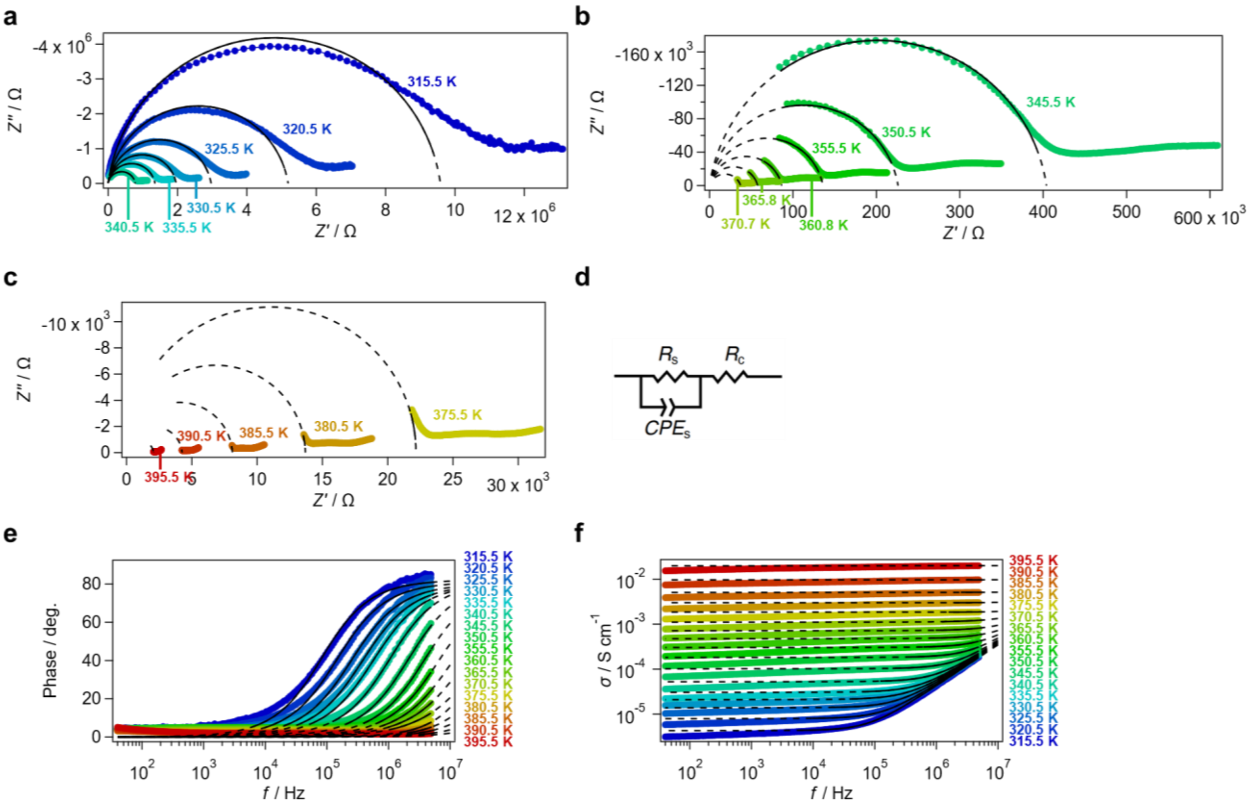

Figure S16. Frequency-dependent AC impedance data for the single crystal of 1-d_2 measured along the *b*-axis. a–**c**, Cole–Cole plots at different temperatures. **d**, Equivalent circuit used for fitting analyses (*R*s: sample resistance, *R*c: contact resistance, *CPE*s: constant phase element for the samples). **e**, **f**, Bode plots at different temperatures. Colored circles: experimental data, solid curves: fitting curves assuming the equivalent circuit shown in **d**, dashed curves: extrapolated curves assuming the same equivalent circuit.

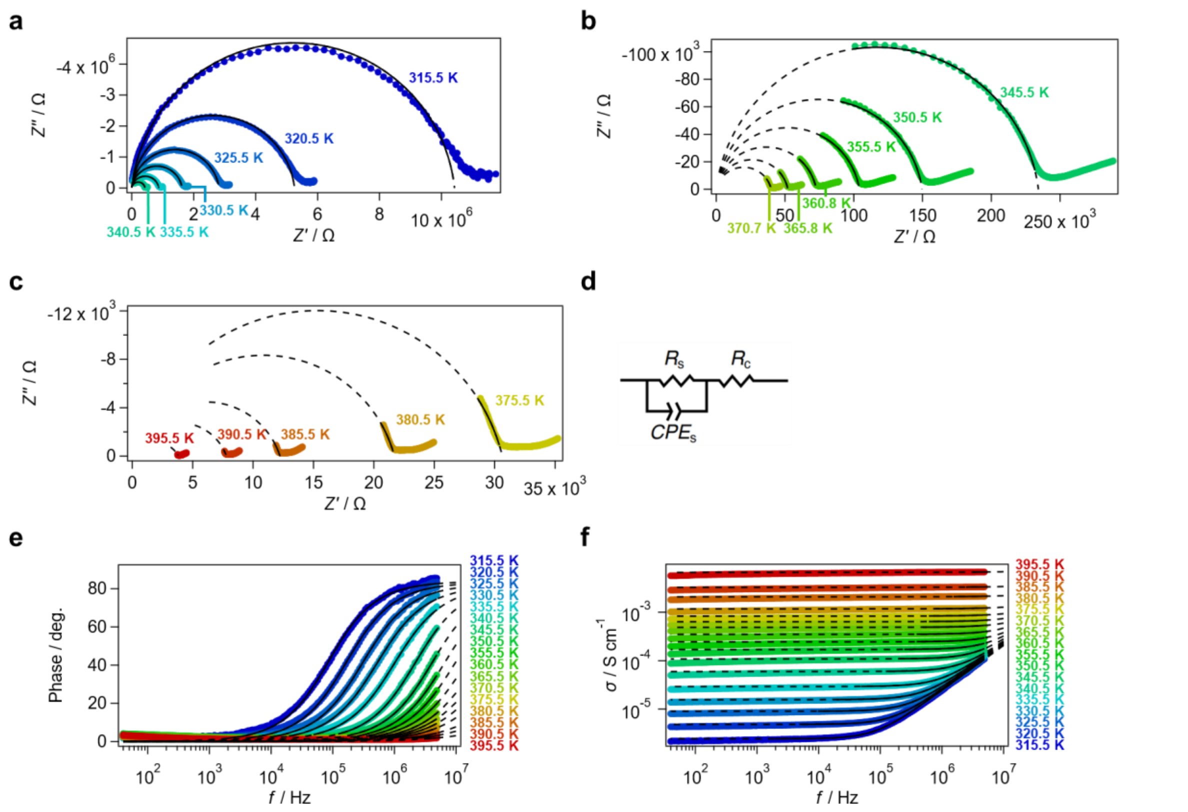

Figure S17. Frequency-dependent AC impedance data for the single crystal of 1-d_2 measured along the *c*-axis. a−**c**, Cole–Cole plots at different temperatures. **d**, Equivalent circuit used for fitting analyses (*R*s: sample resistance, *R*c: contact resistance, *CPE*s: constant phase element for the samples). **e**, **f**, Bode plots at different temperatures. Colored circles: experimental data, solid curves: fitting curves assuming the equivalent circuit shown in **d**, dashed curves: extrapolated curves assuming the same equivalent circuit.

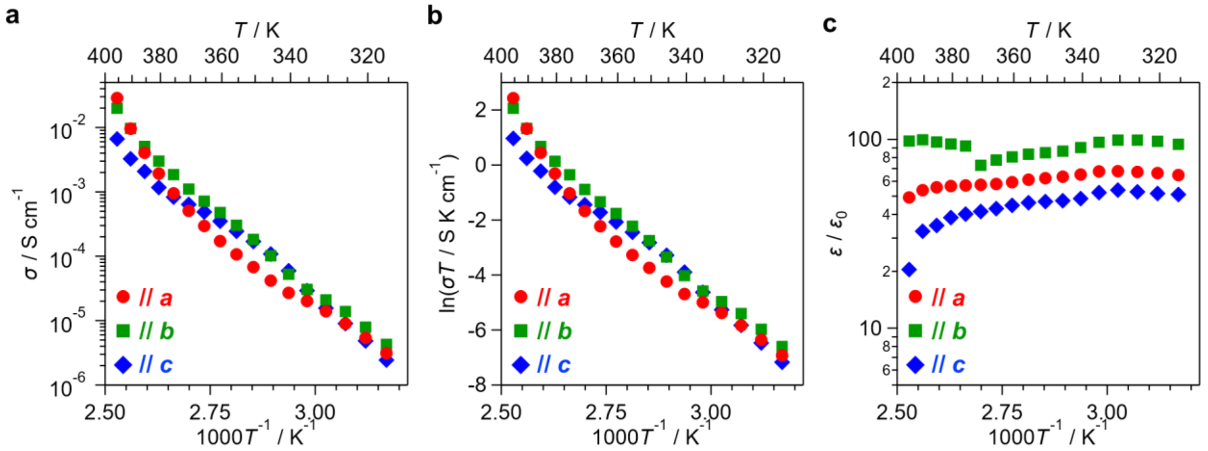

Figure S18. a, Temperature dependence of anhydrous proton conductivity (σ) and b, Arrhenius plots of the anhydrous proton conductivity, and c, temperature dependence of relative permittivity of 1-d_2. Red circles: along the *a*-axis, green squares: along the *b*-axis, blue diamonds: along the *c*-axis.

Table S2 Maximum anhydrous proton conductivity, σ_{max}, and activation energy, E_a, of single-crystal samples of 1-d_2 for each crystallographic direction

	// *a*	// *b*	// *c*
σ_{max} / S cm^{-1}	2.86 × 10^{-2}	1.98 × 10^{-2}	6.62 × 10^{-3}
	[395.5 K]	[395.5 K]	[395.5 K]
E_a / eV	1.16(6)	1.13(2)	1.03(2)
	[315.5−395.5 K]	[315.5−395.5 K]	[315.5−395.5 K]

Demonstration of fuel-cell device using single crystals of 1

Although the single crystals of **1** showed superprotonic conductivity in AC impedance measurements, this measurement technique alone cannot prove H^+ is the conducting carrier. To obtain direct evidence that **1** can efficiently conduct H^+, fuel-cell devices using the single crystals were fabricated, and a digital clock was powered by the device to demonstrate the proton-conducting property of **1**. When five single crystals of **1** were first connected in series, a high electromotive force of about 3.3 V was achieved (Figure S19a), but the clock did not run at room temperature because the internal resistance of 1.4 MΩ was much higher than the resistance of the clock (0.2 MΩ). To reduce the internal resistance of the fuel cell, the crystals were heated with a heat gun, allowing the clock to run at approximately 383 K, although the clock dials flickered and did not operate stably. Therefore, the circuit was reconfigured to minimize the effect of internal resistance. Three, four, and four crystals were first connected in series, and then these three series were connected in parallel (Figure S19b). The improved fuel cell was able to run the clock even at room temperature, albeit unstably, and operated stably at 320 K. This result clearly proved that **1** can efficiently conduct H^+; **1** is an anhydrous superprotonic conductor.

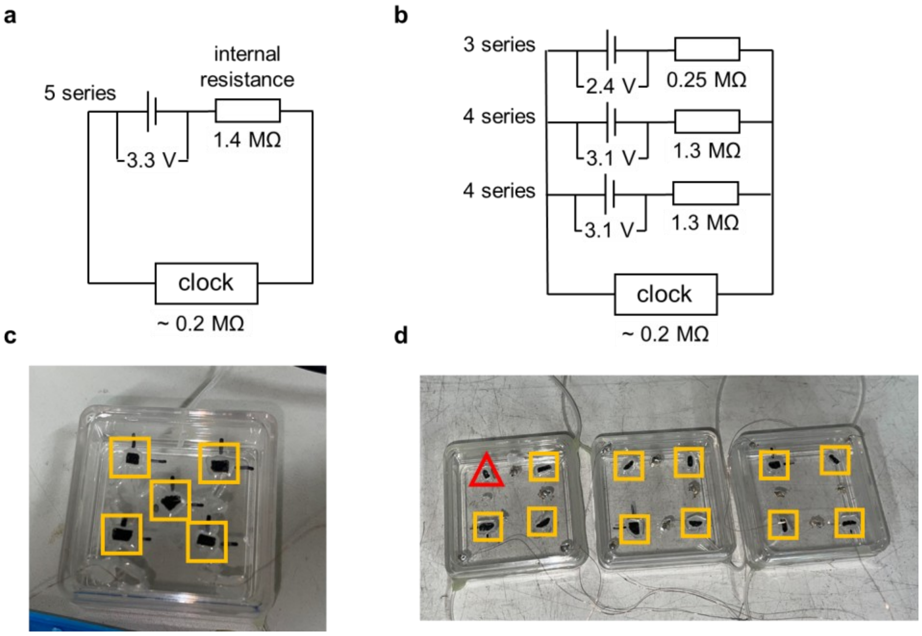

Figure S19. Fuel-cell device using the single crystal of 1 as an electrolyte. a, **b**, Circuits of the fabricated fuel cell connected to a clock. The electromotive force and resistance were measured simply using a tester at room temperature. **a**, Five single crystals of **1** attached with electrodes were connected in series. **b**, Three, four, and four single crystals of **1** were connected in series, and these three series were connected in parallel. **c**, **d**, Images of the fuel cells corresponding to **a**, and **b**, respectively. The single crystals of **1** are framed by orange squares, and a crystal that broke and was not incorporated into the circuit is framed by a red triangle.

Structural change of 1,2,3-TrzH$^+$ via tautomerism by varying temperature

The change in bond length accompanied by proton tautomerism of **1,2,3-TrzH$^+$** was estimated by first-principles calculations (Figure S20). The following optimized structures after structural optimizations were considered: (1) all the four **1,2,3-TrzH$^+$** cations in the unit cell are **1*H*,3*H*-1,2,3-TrzH$^+$** (Figure S20a, center), (2) one of the four **1,2,3-TrzH$^+$** becomes **2*H*-1,2,3-Trz** (Figure S20a, right). While the change from model (1) to model (2) corresponds to proton conduction to the direction of the −*b* axis, proton conduction to the direction of the +*b* axis should occur as well. Therefore, an additional calculation was performed to obtain the optimized structure (3), in which the proton attached to nitrogen N1 is moved to N2, resulting in one of the four **1,2,3-TrzH$^+$** cations becoming **2*H*,3*H*-1,2,3-TrzH$^+$** (Figure S20a, left). The calculated bond lengths of **1*H*,3*H*-1,2,3-TrzH$^+$** in model (1) and the average bond lengths of **2*H*-1,2,3-Trz** in model (2) and **2*H*,3*H*-1,2,3-TrzH$^+$** in model (3) are shown in Figure S20c. The lengths of the N1−N2, N2−N3, and C1−C2 bonds are expected to increase, while the lengths of N1−C2 and N3−C1 bonds are expected to decrease as the proportion of **2*H*-1,2,3-Trz** or **2*H*,3*H*-1,2,3-TrzH$^+$** increases due to proton tautomerism. This change in bond length corresponds to the N−N and C−C bonds becoming single-bond-like and the C−N bonds becoming double-bond-like due to tautomerism.

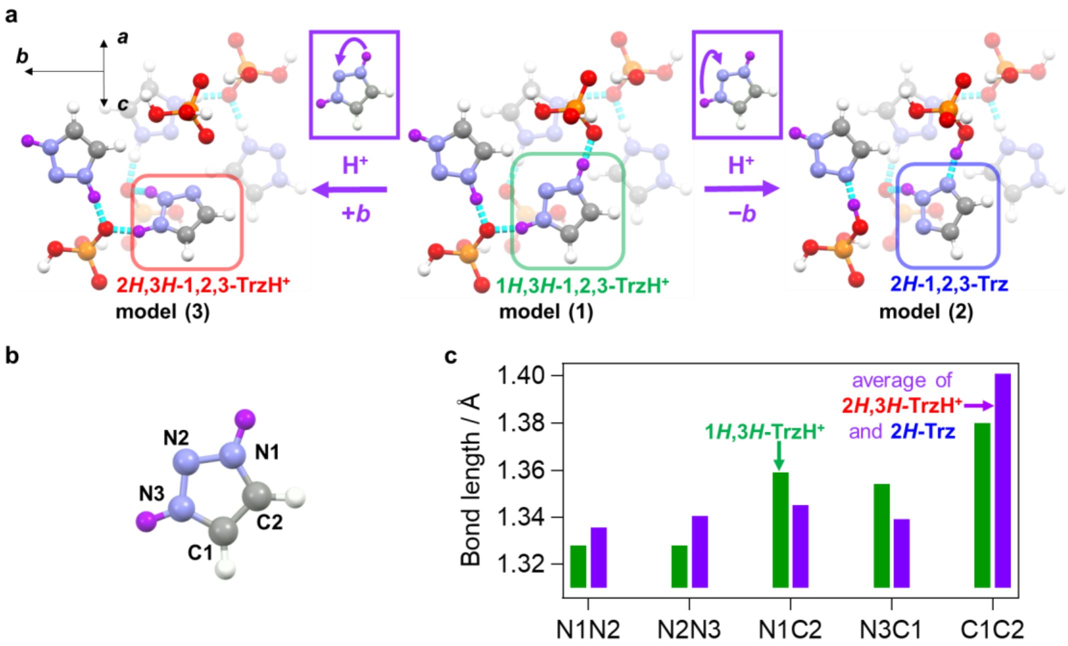

Figure S20. Theoretical prediction of structural change of 1,2,3-TrzH$^+$. a, Structural models of **1** to estimate the change in bond lengths during proton tautomerism (left: model (3), center: model (1), right: model (2), gray: C, white: H, red: O, blue: N, orange: P, purple: H conducted by tautomerism). Light blue dashed lines correspond to N−H···O and O−H···N hydrogen bonds. **1*H*,3*H*-1,2,3-TrzH$^+$**, **2*H*-1,2,3-Trz**, and **2*H*,3*H*-1,2,3-TrzH$^+$** are highlighted by green, blue, and red frames, respectively. Purple arrows correspond to the direction of proton transfer. **b**, Atomic labels in **1,2,3-TrzH$^+$**. **c**, Bond lengths of **1*H*,3*H*-1,2,3-TrzH$^+$** in model (1) (green) and the average bond lengths of **2*H*-1,2,3-Trz** in model (2) and **2*H*,3*H*-1,2,3-TrzH$^+$** in model (3) (purple), obtained by structural optimization.

Variable-temperature single-crystal XRD measurements of **1** were performed at 313, 323, 333, 343, 353, 363, and 373 K. Structural analysis at each temperature revealed that the crystal system and space group remained unchanged at all temperatures, and that the crystal axes length and unit cell volume increased uniformly (Table S3).

Table S3 Crystallographic data of 1 from variable-temperature XRD structural analysis

Temperature / K	313.0	323.0	333.0	343.0	353.0	363.0	373.0
Empirical formula	$C_2H_6N_3O_4P$	$C_2H_6N_3O_4P$	$C_2H_6N_3O_4P$	$C_2H_6N_3O_4P$	$C_2H_6N_3O_4P$	$C_2H_6N_3O_4P$	$C_2H_6N_3O_4P$
Crystal system	monoclinic	monoclinic	monoclinic	monoclinic	monoclinic	monoclinic	monoclinic
Space group	$P2_1/c$	$P2_1/c$	$P2_1/c$	$P2_1/c$	$P2_1/c$	$P2_1/c$	$P2_1/c$
a / Å	9.2713(7)	9.2735(7)	9.2842(7)	9.2891(5)	9.3024(4)	9.3060(4)	9.3031(6)
b / Å	9.7707(7)	9.7731(6)	9.7742(7)	9.7765(5)	9.7798(5)	9.7790(5)	9.7798(6)
c / Å	7.1317(5)	7.1338(5)	7.1418(5)	7.1461(4)	7.1459(4)	7.1483(4)	7.1535(5)
β / °	92.334(7)	92.286(6)	92.146(7)	92.103(5)	91.992(5)	91.913(5)	91.870(6)
Volume / Å^3	645.50(8)	646.03(8)	647.63(8)	648.54(6)	649.71(6)	650.16(6)	650.50(7)
Z value	4	4	4	4	4	4	4
Z' value	1	1	1	1	1	1	1
R_1 (I >= 2.00σ(I))	0.0330	0.0326	0.0351	0.0381	0.0388	0.0415	0.0690
wR_2 factor (all)	0.0894	0.0904	0.0965	0.1035	0.1108	0.1220	0.2350
Goodness of fit	1.015	1.054	1.073	1.059	1.104	1.109	1.148
CCDC	2471905	2476509	2471906	2471908	2471918	2471919	2471920

Quantum chemical calculations of a single molecule of 1,2,3-TrzH$^+$

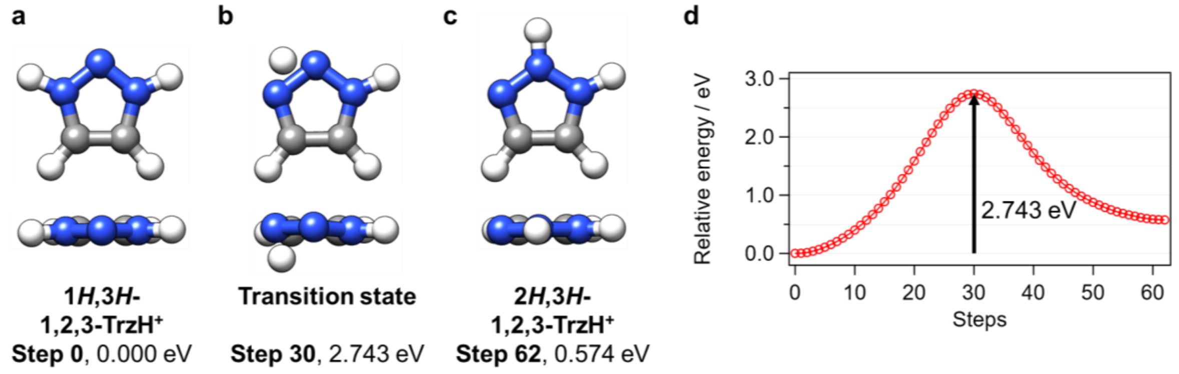

Figure S21. Structures and potential energy of a single molecule of 1,2,3-TrzH$^+$. a, **1*H*,3*H*-1,2,3-TrzH$^+$**, **b**, transition state, and **c**, **2*H*,3*H*-1,2,3-TrzH$^+$** (top: top view, bottom: side view; gray: C, white: H, blue: N). **d**, Potential energy curve obtained from IRC calculation (the lowest value at Step 0 was set to 0).

Perturbation energy ($-\mu \cdot \epsilon$) in *ab initio* NEMD

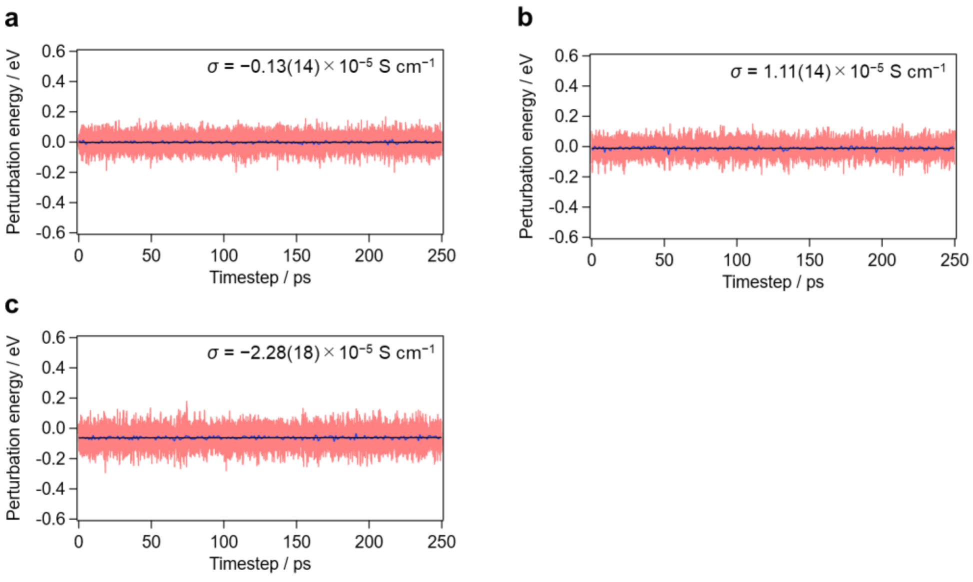

Figure S22. Perturbation energy ($-\mu \cdot \epsilon$) of the perfect crystal in *ab initio* NEMD.

The electrostatic field was applied along **a,** the *a*-axis: (1.000×10^9, 0, 0) V/m, **b,** the *b*-axis: (0, 1.000×10^9, 0) V/m, and **c,** the *c*-axis: (4.164×10^7, 0, 9.991×10^8) V/m. Red: plots per 0.25 fs, blue: averaged plots per 1 ps, black: regression line. σ was determined by the slope of the regression line as derived above.

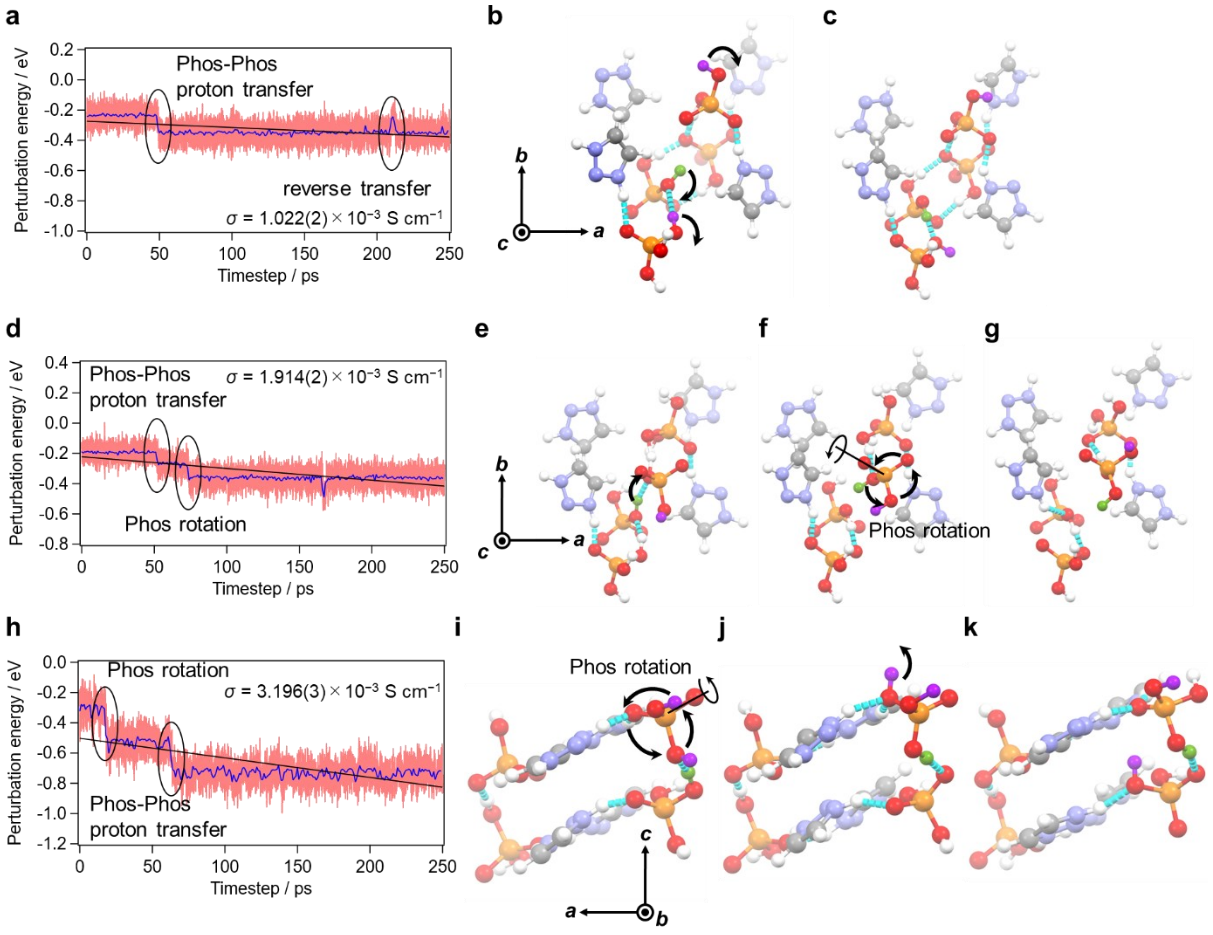

Figure S23. Perturbation energy (−μ · ϵ) of the proton-doped crystal in *ab initio* NEMD and the structures before and after significant atomic displacement.

The electrostatic field was applied along **a,** the *a*-axis: (1.000×10^9, 0, 0) V/m, **d,** the *b*-axis: (0, 1.000×10^9, 0) V/m, and **h,** the *c*-axis: (4.164×10^7, 0, 9.991×10^8) V/m. Red: plots per 0.25 fs, blue: averaged plots per 1 ps, black: regression line. The point at which the atoms moved significantly is circled. σ was determined by the slope of the regression line as derived above. Structures with the electrostatic field along the *a*-axis at **b,** 48.12 ps and **c,** 50.74 ps. Structures with the electrostatic field along the *b*-axis at **e,** 52.44 ps, **f,** 63.00 ps, and **g,** 74.91 ps. Structures with the electrostatic field along the *c*-axis at **i,** 16.95 ps, **j,** 43.63 ps, and **k,** 64.58 ps. Gray: C, white: H, blue: N, red: O, orange: P, green: doped proton, purple: significantly moved proton. Black curved arrow indicates the movement of atoms.

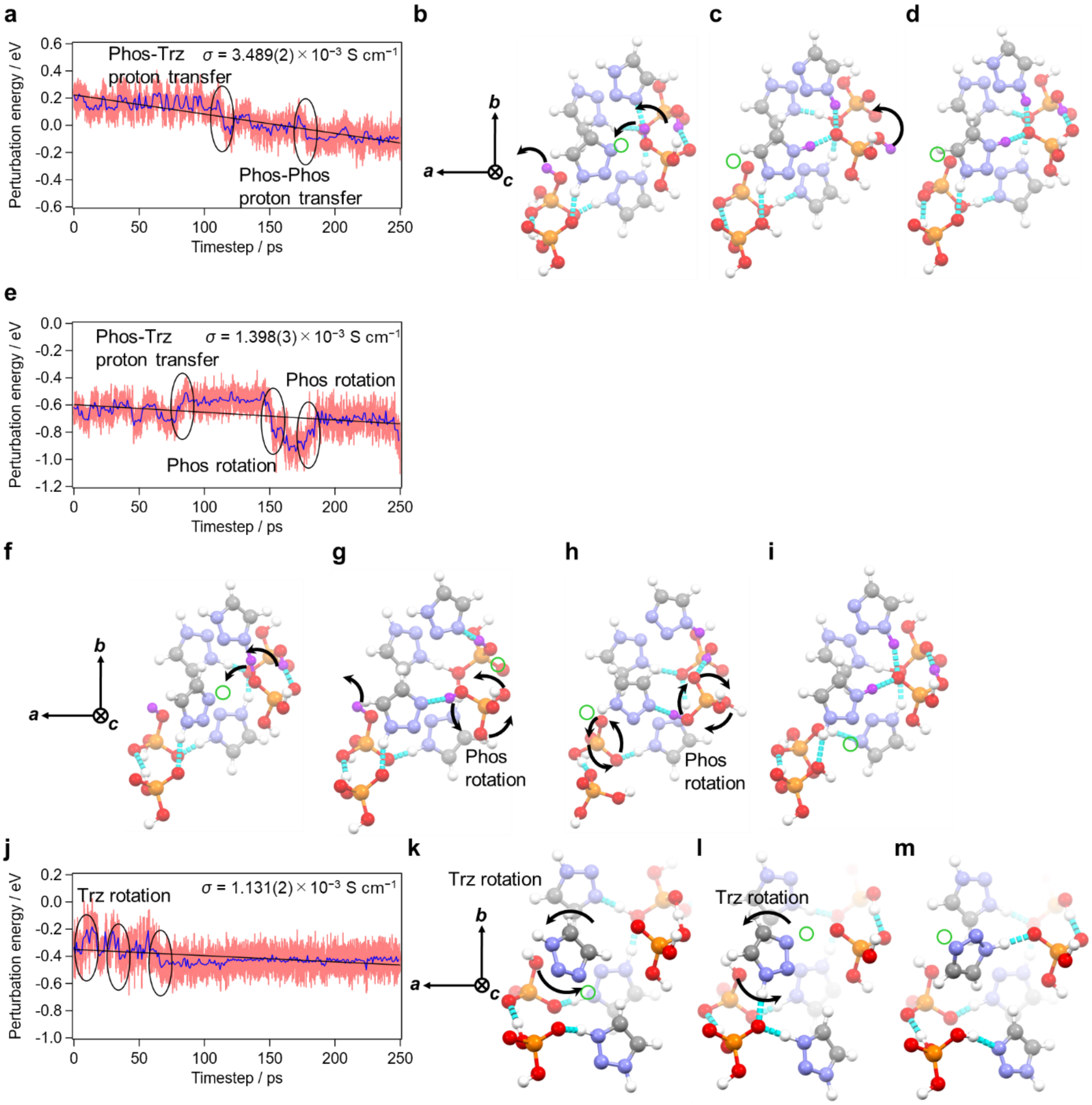

Figure S24. Perturbation energy ($-\mu \cdot \epsilon$) of the defect-doped crystal in *ab initio* NEMD and the structures before and after significant atomic displacement.

The electrostatic field was applied along **a,** the *a*-axis: (1.000×10^9, 0, 0) V/m, **e,** the *b*-axis: (0, 1.000×10^9, 0) V/m, and **j,** the *c*-axis: (4.164×10^7, 0, 9.991×10^8) V/m. Red: plots per 0.25 fs, blue: averaged plots per 1 ps, black: regression line. The point at which the atoms moved significantly is circled. σ was determined by the slope of the regression line as derived above. Structures with the electrostatic field along the *a*-axis at **b,** 110.92 ps, **c,** 148.46 ps, and **d,** 179.79 ps. Structures with the electrostatic field along the *b*-axis at **f,** 82.75 ps, **g,** 115.92 ps, **h,** 171.51 ps, and **i,** 201.43 ps. Structures with the electrostatic field along the *c*-axis at **k,** 1.08 ps, **l,** 8.22 ps, and **m,** 73.96 ps. Gray: C, white: H, blue: N, red: O, orange: P, green: doped proton, purple: significantly moved proton. Black curved arrow indicates the movement of atoms.

***Ab itnitio* NEB calculation of proton tautomerism**

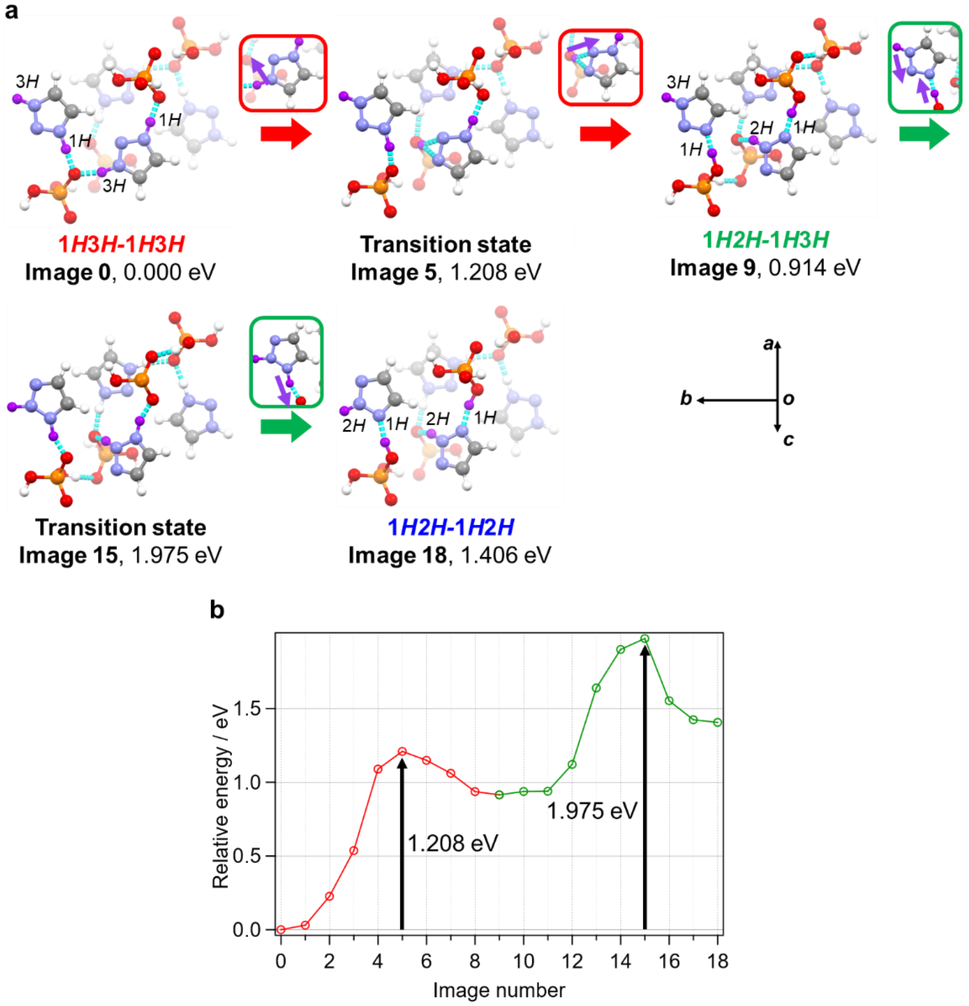

Figure S25. ***Ab initio*** **NEB calculation of proton tautomerism (pathway 1) in the perfect crystal of 1. a,** Structures of local minimum states and transition states (gray: C, white: H, blue: N, red: O, orange: P; the conducting protons are colored purple). NEB calculations were performed for each of the two proton transfer divisions. Images 0, 9, and 18 were optimized individually and correspond to the initial or final states of the respective proton transfer divisions. Images 5 and 15 are the transition states. The corresponding image numbers and relative potential energies (the lowest value for Image 0 was set to 0) are shown beneath the structures. **b**, Relative energy curve obtained from NEB calculation for tautomerism process of **1** (red: the first division, green: the second division).

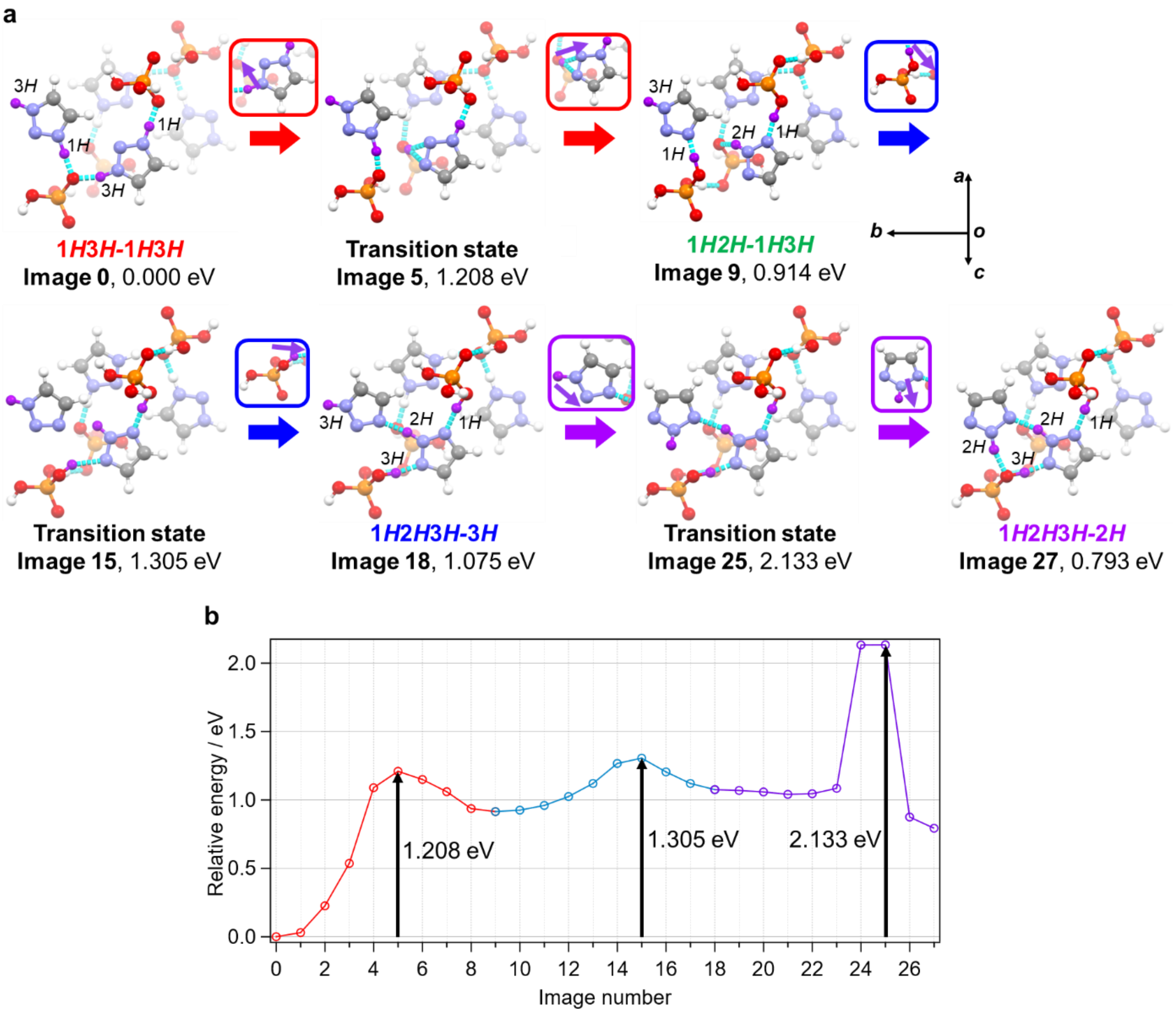

Figure S26. ***Ab initio*** **NEB calculation of proton tautomerism (pathway 2) in the perfect crystal of 1.** **a,** Structures of local minimum states and transition states (gray: C, white: H, blue: N, red: O, orange: P; the conducting protons are colored purple). NEB calculations were performed for each of the three proton transfer divisions. Images 0, 9, 18, and 27 were optimized individually and correspond to the initial or final states of the respective proton transfer divisions. Images 5, 15, and 25 are the transition states. The corresponding image numbers and relative potential energies (the lowest value for Image 0 was set to 0) are shown beneath the structures. **b**, Relative energy curve obtained from NEB calculation for tautomerism process of **1** (red, blue, purple: the first, second, and third divisions, respectively).

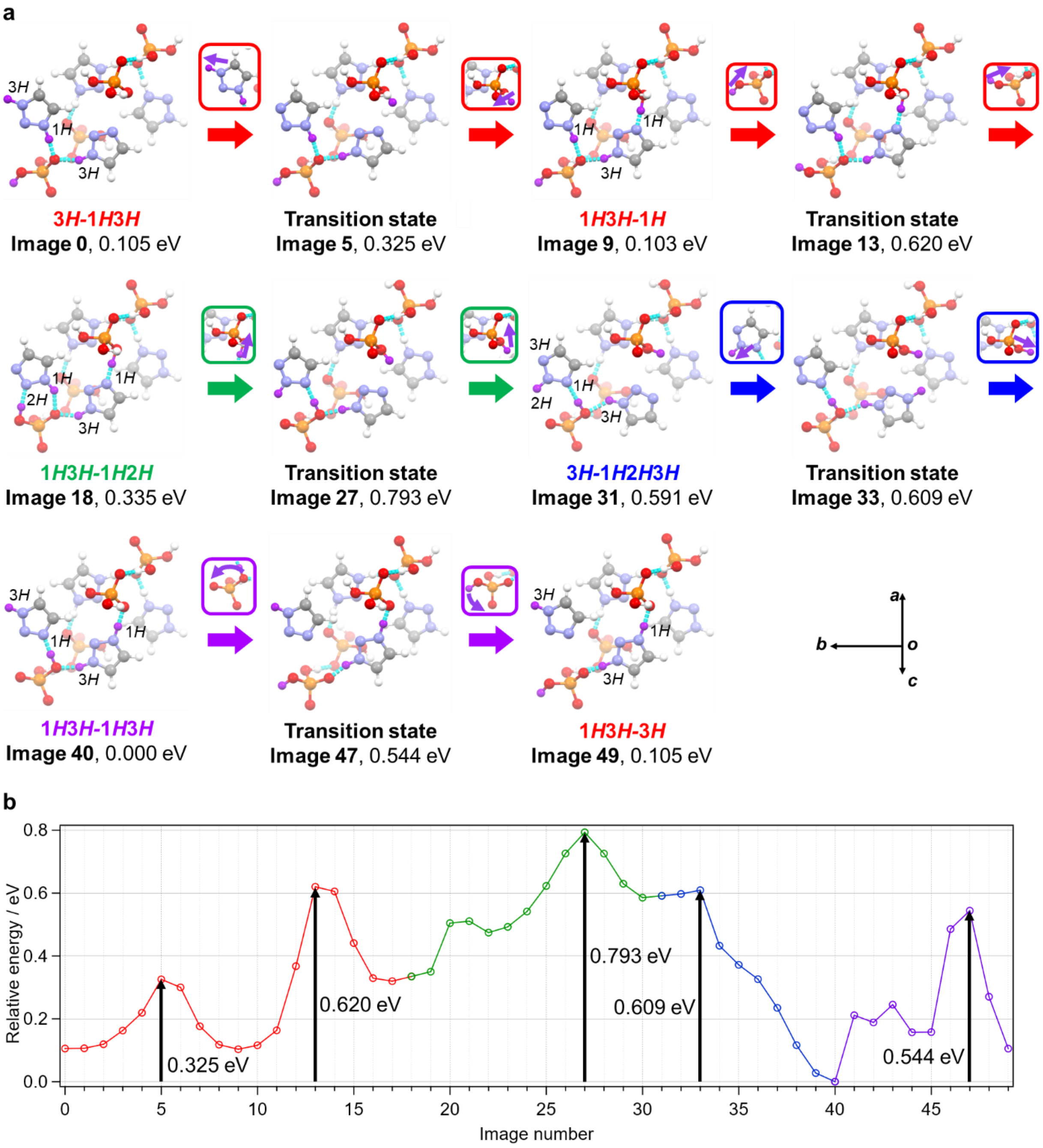

Figure S27. ***Ab initio*** **NEB calculation of proton tautomerism in the crystal of 1 with proton defects.** **a,** Structures of local minimum states and transition states (gray: C, white: H, blue: N, red: O, orange: P; the conducting protons are colored purple). NEB calculations were performed for each of the three proton transfer divisions. Images 0, 9, 18, 31, 40, and 49 were optimized individually and correspond to the initial or final states of the respective proton transfer divisions. Images 5, 13, 27, 33, and 47 are the transition states. The corresponding image numbers and relative potential energies (the lowest value for Image 40 was set to 0) are shown beneath the structures. **b**, Relative energy curve obtained from NEB calculation for tautomerism process of **1** (red, green, blue, purple: the first, second, third, and fourth divisions, respectively).

References

[1] A. Abbotto, S. Bradamante, G. A. Pagani, “Diheteroarylmethanes. 5.1 E−Z Isomerism of Carbanions Substituted by 1,3-Azoles: ^{13}C and ^{15}N π-Charge/Shift Relationships as Source for Mapping Charge and Ranking the Electron-Withdrawing Power of Heterocycles” *J. Org. Chem.* **1996**, *61*, 1761–1769.

[2] O. V. Dolomanov, L. J. Bourhis, R. J. Gildea, J. a. K. Howard, H. Puschmann, “OLEX2: a complete structure solution, refinement and analysis program” *J. Appl. Crystallogr.* **2009**, *42*, 339–341.

[3] F. H. Larsen, H. J. Jakobsen, P. D. Ellis, N. Chr. Nielsen, “Molecular dynamics from ^{2}H Quadrupolar Carr–Purcell–Meiboom–Gill solid-state NMR spectroscopy” *Chem. Phys. Lett.* **1998**, *292*, 467–473.

[4] M. Shiga, “PIMD,” can be found under https://ccse.jaea.go.jp/software/PIMD/index.en.html.

[5] T. D. Kühne et al., “CP2K: An electronic structure and molecular dynamics software package - Quickstep: Efficient and accurate electronic structure calculations” *J. Chem. Phys.* **2020**, *152*, 194103.

[6] B. Hammer, L. B. Hansen, J. K. Nørskov, “Improved adsorption energetics within density-functional theory using revised Perdew-Burke-Ernzerhof functionals” *Phys. Rev. B* **1999**, *59*, 7413–7421.

[7] M. A. L. Marques, M. J. T. Oliveira, T. Burnus, “LIBXC: A library of exchange and correlation functionals for density functional theory” *Comput. Phys. Commun.* **2012**, *183*, 2272–2281.

[8] S. Lehtola, C. Steigemann, M. J. T. Oliveira, M. A. L. Marques, “Recent developments in libxc — A comprehensive library of functionals for density functional theory” *SoftwareX* **2018**, *7*, 1–5.

[9] J. VandeVondele, J. Hutter, “Gaussian basis sets for accurate calculations on molecular systems in gas and condensed phases” *J. Chem. Phys.* **2007**, *127*, 114105.

[10] S. Goedecker, M. Teter, J. Hutter, “Separable dual-space Gaussian pseudopotentials” *Phys. Rev. B* **1996**, *54*, 1703–1710.

[11] S. Grimme, J. Antony, S. Ehrlich, H. Krieg, “A consistent and accurate ab initio parametrization of density functional dispersion correction (DFT-D) for the 94 elements H-Pu” *J. Chem. Phys.* **2010**, *132*, 154104.

[12] S. Grimme, S. Ehrlich, L. Goerigk, “Effect of the damping function in dispersion corrected density functional theory” *J. Comput. Chem.* **2011**, *32*, 1456–1465.

[13] A. Marek et al., “The ELPA library: scalable parallel eigenvalue solutions for electronic structure theory and computational science” *J. Phys. Condens. Matter* **2014**, *26*, 213201.

[14] M. Frigo, S. G. Johnson, “The Design and Implementation of FFTW3” *Proc. IEEE* **2005**, *93*, 216–231.

[15] D. J. Evans, G. Morriss, *Statistical Mechanics of Nonequilibrium Liquids*, Cambridge University Press, Cambridge, **2008**.

[16] D. J. Evans, W. G. Hoover, B. H. Failor, B. Moran, A. J. C. Ladd, “Nonequilibrium molecular dynamics via Gauss’s principle of least constraint” *Phys. Rev. A* **1983**, *28*, 1016–1021.

[17] P. C. Aeberhard, S. R. Williams, D. J. Evans, K. Refson, W. I. F. David, “Ab initio Nonequilibrium Molecular Dynamics in the Solid Superionic Conductor ${\mathrm{LiBH}}_{4}$” *Phys. Rev. Lett.* **2012**, *108*, 095901.

[18] T. Ozaki, “Variationally optimized atomic orbitals for large-scale electronic structures” *Phys. Rev. B* **2003**, *67*, 155108.

[19] T. Ozaki, H. Kino, "Numerical atomic basis orbitals from H to Kr" *Phys. Rev. B* **2004**, *69*, 195113.
[20] T. Ozaki et al., "OpenMX," can be found under https://www.openmx-square.org/index.html.
[21] K. Lejaeghere et al., "Reproducibility in density functional theory calculations of solids" *Science* **2016**, *351*, aad3000.
[22] T. Ozaki, H. Kino, "Efficient projector expansion for the ab initio LCAO method" *Phys. Rev. B* **2005**, *72*, 045121.
[23] J. P. Perdew, K. Burke, M. Ernzerhof, "Generalized Gradient Approximation Made Simple" *Phys. Rev. Lett.* **1996**, *77*, 3865–3868.
[24] G. Henkelman, H. Jónsson, "Improved tangent estimate in the nudged elastic band method for finding minimum energy paths and saddle points" *J. Chem. Phys.* **2000**, *113*, 9978–9985.
[25] Gaussian 16, Revision C.01, M. J. Frisch, et. al., Gaussian, Inc., Wallingford CT, **2016.**
[26] Y. Zhao, D. G. Truhlar, "The M06 suite of density functionals for main group thermochemistry, thermochemical kinetics, noncovalent interactions, excited states, and transition elements: two new functionals and systematic testing of four M06-class functionals and 12 other functionals" *Theor. Chem. Acc.* **2008**, *120*, 215–241.
[27] C. Gonzalez, H. B. Schlegel, "An improved algorithm for reaction path following" *J. Chem. Phys.* **1989**, *90*, 2154–2161.
